# Supplementary material for: Stable and oxidative charged Ru enhance the acidic oxygen evolution reaction activity in two-dimensional ruthenium-iridium oxide
Source: Nat Commun. 2023 Sep 4;14:5365. doi: 10.1038/s41467-023-41036-9 (PMC10477217; doi:10.1038/s41467-023-41036-9)
Supplement: Supplementary file 1 — Supplementary Information [file 41467_2023_41036_MOESM1_ESM.pdf]

Supporting Information

Stable and oxidative charged Ru enhance the acidic oxygen evolution reaction activity in two-dimensional ruthenium-iridium oxide

Wenxiang Zhu[1,†], Xiangcong Song[1,†], Fan Liao[1], Hui Huang[1], Qi Shao[2], Kun Feng[1], Yunjie Zhou[1], Mengjie Ma[1], Jie Wu[1], Hao Yang[1], Haiwei Yang[1], Meng Wang[1], Jie Shi[1], Jun Zhong[1], Tao Cheng[1,*], Mingwang Shao[1,*], Yang Liu[1,*] and Zhenhui Kang[1,3,*]

[1]Institute of Functional Nano & Soft Materials (FUNSOM), Jiangsu Key Laboratory for Carbon-Based Functional Materials & Devices, Soochow University, 199 Ren'ai Road, Suzhou, 215123, Jiangsu, China.

[2]College of Chemistry, Chemical Engineering and Materials Science, Soochow University, Jiangsu 215123, China.

[3]Macao Institute of Materials Science and Engineering (MIMSE), MUST-SUDA Joint Research Center for Advanced Functional Materials, Macau University of Science and Technology, Taipa 999078, Macao, China.

[†]These authors contributed equally: Wenxiang Zhu and Xiangcong Song

*To whom correspondence should be addressed.

E-mail: tcheng@suda.edu.cn; mwshao@suda.edu.cn; yangl@suda.edu.cn; zhkang@suda.edu.cn

Details of density functional theory (DFT) calculations.

Entropy Correction and solvation correction

The frequencies and normal modes are determined from the Hessian matrix. To calculate the Hessian matrix, finite differences are used. Each ion is displaced ± 0.04 A in the direction of each cartesian coordinate, and from the forces the Hessian matrix is determined. Only the adsorbed species are populated in the frequency calculation, while the slab atoms were kept fixed. The temperature is 298 K, and the pressure is 1 bar.

Free energies for all structures were computed using standard statistical mechanics formula that accounts for translational, rotational, vibrational, and electronic degrees of freedom. Translational and rotational contributions to the free energy were omitted for all surface adsorbed species. The estimation of the thermodynamics is briefly summarized as follows.

The partition function under the harmonic oscillator approximation is as follows:

$$q_{\mathrm{vib}} = \prod_i \frac{e^{-hv_i/2kT}}{1 - e^{-hv_i/kT}} \quad (1)$$

Where k is a force constant, v is the vibrational frequency. After substituting the partition function q_{vib}, the internal energy correction is as follows:

$$U_{\mathrm{vib}}(T) = R\sum_i \left(\frac{hv_i}{k}\right)\left(\frac{1}{2} + \frac{e^{-\frac{hv_i}{kT}}}{1 - e^{-\frac{hv_i}{kT}}}\right) (2)$$

The first term is the contribution of zero-point energy (ZPE), and the second term is the contribution of internal energy correction from 0 K to 298K.

The correction of entropy (S) is as follows:

$$S_{\mathrm{vib}}(T) = R\sum_i \left\{\frac{hv_i}{kT}\frac{e^{-\frac{hv_i}{kT}}}{1 - e^{-\frac{hv_i}{kT}}} - \ln\left[1 - e^{-\frac{hv_i}{kT}}\right]\right\} (3)$$

Here, the first term exactly cancels with the second term of the internal energy shown above. Therefore, only the second term of the entropy needs to be corrected.

For adsorbed species, the six degrees of freedom of the translation and rotation are frustrated and considered vibration. Such approximation fails when the vibration is extremely low, which has a significant contribution to the correction. To avoid such overestimation, the contribution of frequencies below 50 cm^{-1} are all considered as 50 cm^{-1}.

Zero-point energy (ZPE) in thermo energy correction is as follows:

$$\varepsilon_{\mathrm{ZPE}} = \frac{hv}{2} \quad (4)$$

The Gibbs free energy G can be derived as follows:

$$G = E_{ZPE} + E + k_B T - TS \ (5)$$

Finally, Implicit solvation is taken into account[1,2]:

$$G_{sol} = E_{ZPE} + E_{sol} + k_B T - TS \ (6)$$

To include solvation effects, we used an implicit solvation model to describe the effect of electrostatics and dispersion on the interaction between the surface catalyst structure and solvent, as implemented VASP sol[1,2] (patched into VASP 5.4.4). The dielectric constant was set to 78.4, corresponding to water at room temperature. Single-point solvation corrections were applied to the optimized structures:

$$G_{sol} = E_{ZPE} + E_{sol} + k_B T - TS \ (7)$$

The OER reaction steps in the acidic solution and the calculated OER overpotential are as following:

$$H_2O + * \rightarrow * OH + H^+ + e^- \quad (8)$$

$$\Delta G_1 = G(*OH) + \frac{1}{2}G(H_2) - G(H_2O) - G(*) \quad (9)\#$$

$$*OH \rightarrow O* + H^+ + e^- \quad (10)$$

$$\Delta G_2 = G(O*) + \frac{1}{2}G(H_2) - G(*OH) \quad (11)\#$$

$$O* + H_2O \rightarrow *OOH + H^+ + e^- \quad (12)$$

$$\Delta G_3 = G(*OOH) + \frac{1}{2}G(H_2) - G(O*) - G(H_2O) \quad (13)\#$$

$$*OOH \rightarrow O_2 + * + H^+ + e^- \quad (14)$$

$$\Delta G_4 = 4.92 - \Delta G_1 - \Delta G_2 - \Delta G_3 \quad (15)\#$$

$$\eta = \frac{\max\{\Delta G_1, \Delta G_2, \Delta G_3, \Delta G_4\}}{e} \quad (16)\#$$

The *OOH, *O, and *OH are the OER intermediates absorbed on the catalyst surface.

Fixed potential calculations are performed using the plane wave basis JDFTx code[3] with an energy cutoff of 20 Hartree. Specifies that the exchange association function is perdew-burke-ernzerhof GGA[4]. We consider the effect of the electrochemical potential on the OER reaction energy by a fixed potential calculation with the target-mu command, which directly sets the electronic chemical potential and balances the electron number, exactly as in the electrochemical system. We use the CANDLE model to account for solvation energy effects, which uses SaLSA's non-local electron density overlap method to determine the cavity, but then uses the local dielectric response like other linear PCMS[5].

The solvation energy with fixed-potential is $G = F - mu*N$ where F is the energy and N is the number of electrons, and potential V (in Volts) relative to SHE corresponds to $mu = -(Vref + V)/27.2114$, Vref is the absolute SHE potential in Volts below vacuum (4.44 V).

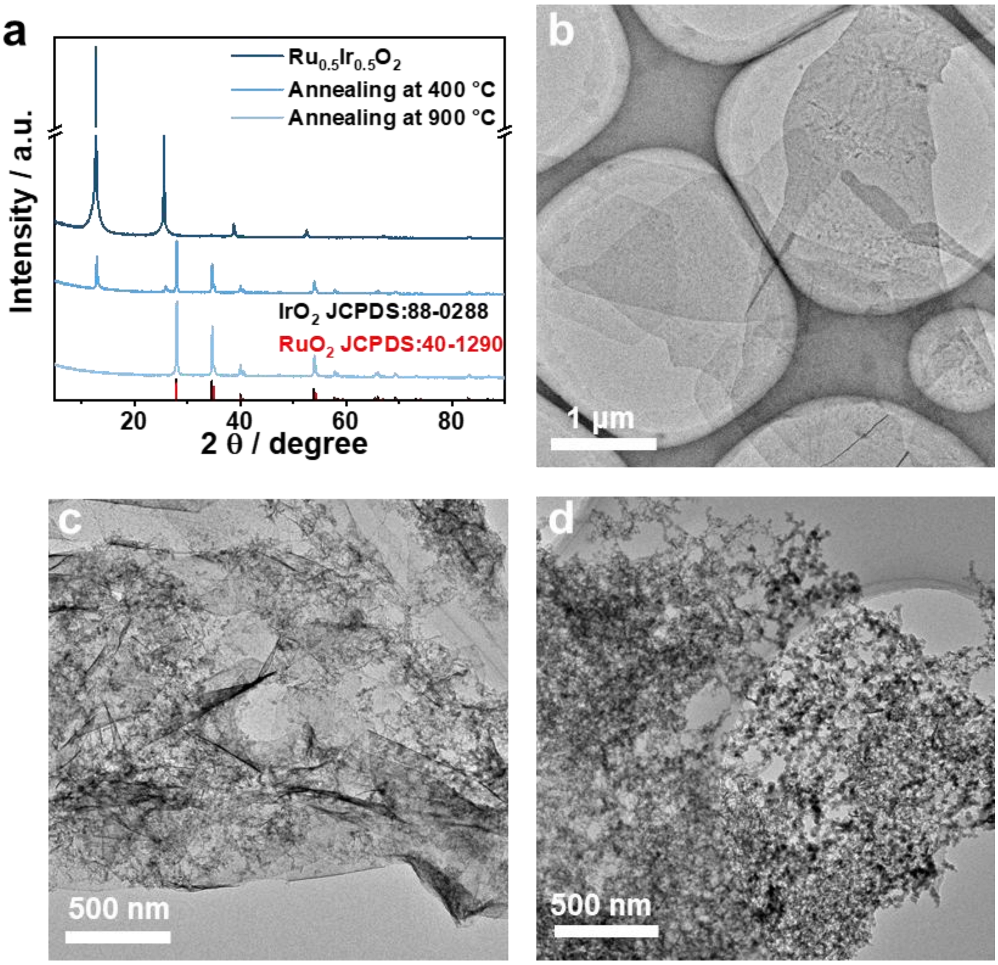

Supplementary Fig. 1 | The XRD patterns and TEM images of $Ru_{0.5}Ir_{0.5}O_2$ annealed at different temperatures. a, Temperature-dependent XRD patterns of annealing $Ru_{0.5}Ir_{0.5}O_2$ at different temperatures. The corresponding TEM images of (**b**) $Ru_{0.5}Ir_{0.5}O_2$ and the products by annealing $Ru_{0.5}Ir_{0.5}O_2$ at (**c**) 400 °C and (**d**) 900 °C for 2 hours, respectively.

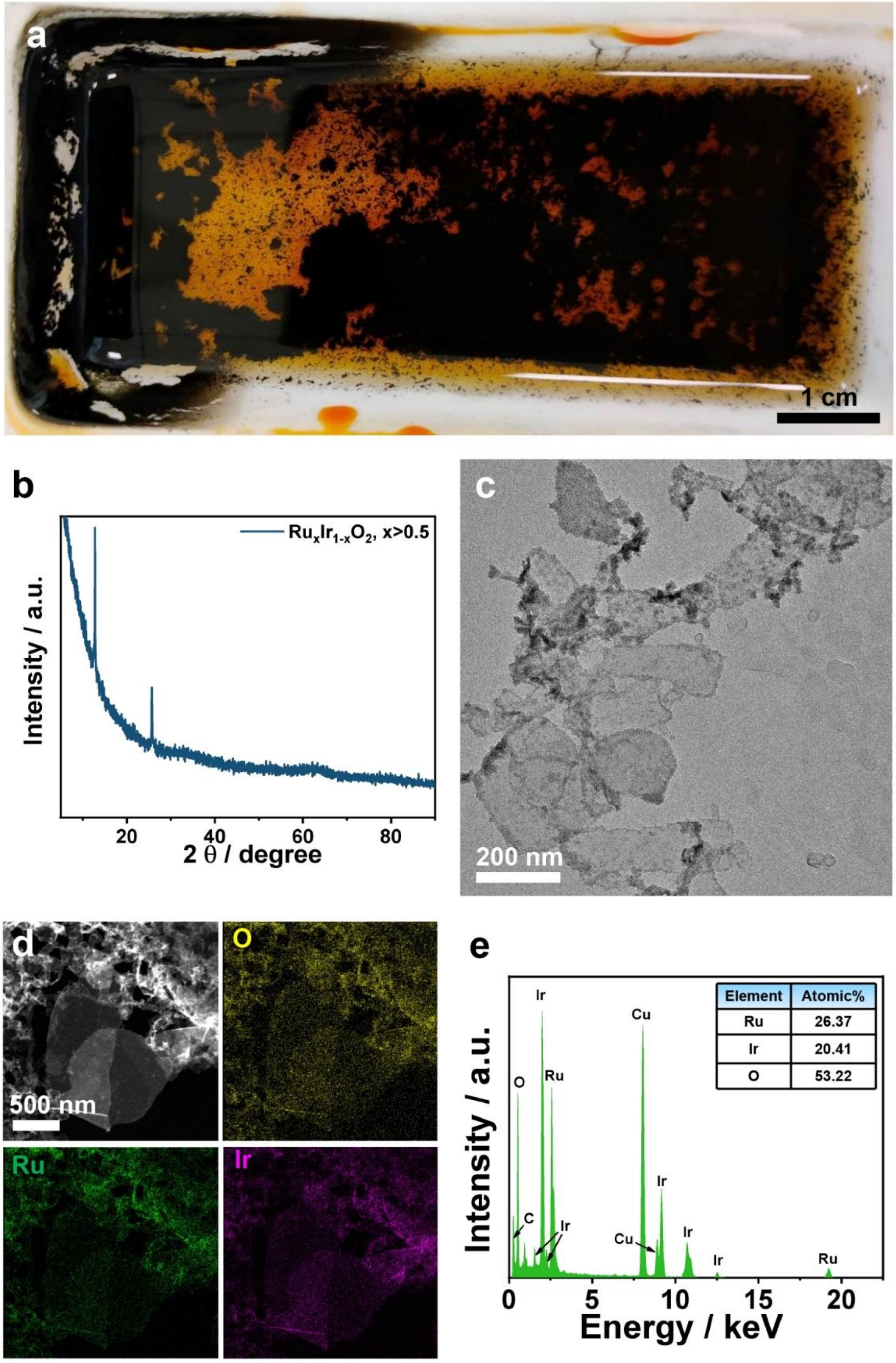

Supplementary Fig. 2 | Characterizations of $Ru_xIr_{1-x}O_2$(x>0.5). a, The obtained $Ru_xIr_{1-x}O_2$(x>0.5) solid product turns orange in the aqueous solution after adding double distilled water, and a large number of soluble substances were dissolved. When the molar amount of raw material ($RuCl_3$) in the second synthesis step was further increased to more than 0.5 M (0.7 M, 145 mg $RuCl_3$), a large amount of soluble Ru/Ir complexes will appear in the obtained product. **b,** XRD pattern of $Ru_xIr_{1-x}O_2$(x>0.5). **c,** TEM image of $Ru_xIr_{1-x}O_2$(x>0.5). **d,** STEM energy-dispersive X-ray spectroscopy (STEM-EDX) mapping of $Ru_xIr_{1-x}O_2$(x>0.5). **e,** TEM-EDX spectrum of $Ru_xIr_{1-x}O_2$(x>0.5).

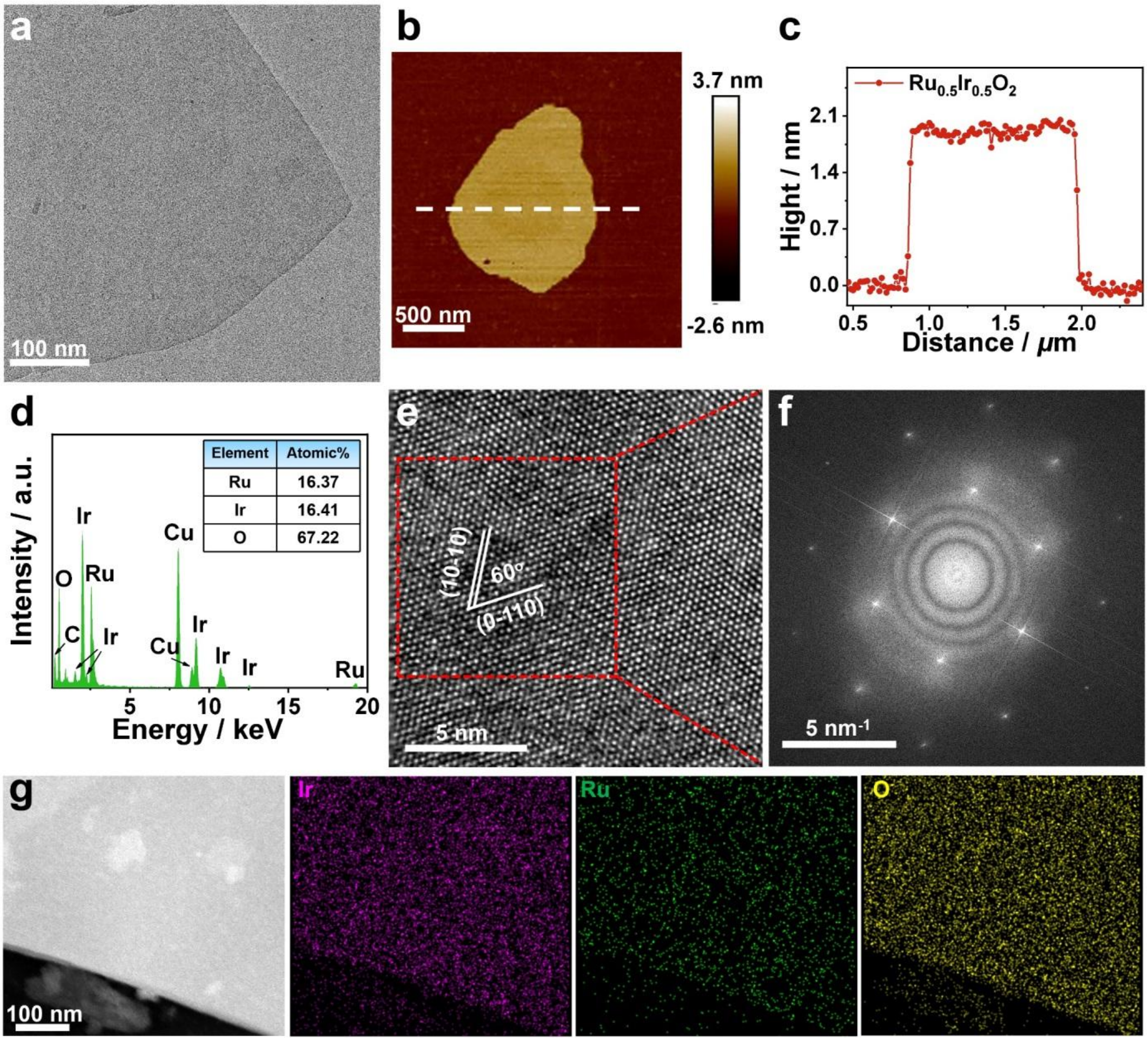

Supplementary Fig. 3 | Characterizations of $Ru_{0.5}Ir_{0.5}O_2$. a, Transmission electron microscopy (TEM) image of $Ru_{0.5}Ir_{0.5}O_2$. **b,** AFM image of $Ru_{0.5}Ir_{0.5}O_2$ and (**c**) the corresponding height profile. **d,** TEM-EDX spectrum of $Ru_{0.5}Ir_{0.5}O_2$. **e,** High resolution transmission electron microscopy (HRTEM) image of $Ru_{0.5}Ir_{0.5}O_2$. **f,** the inset in (**e**) shows the fast Fourier transform (FFT) of the area. **g,** STEM-EDX mapping of $Ru_{0.5}Ir_{0.5}O_2$.

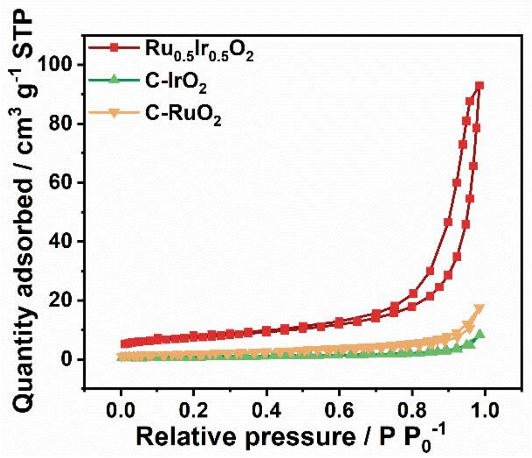

Supplementary Fig. 4 | BET characterization. BET surface area measurements for $Ru_{0.5}Ir_{0.5}O_2$, C-IrO_2 and C-RuO_2.

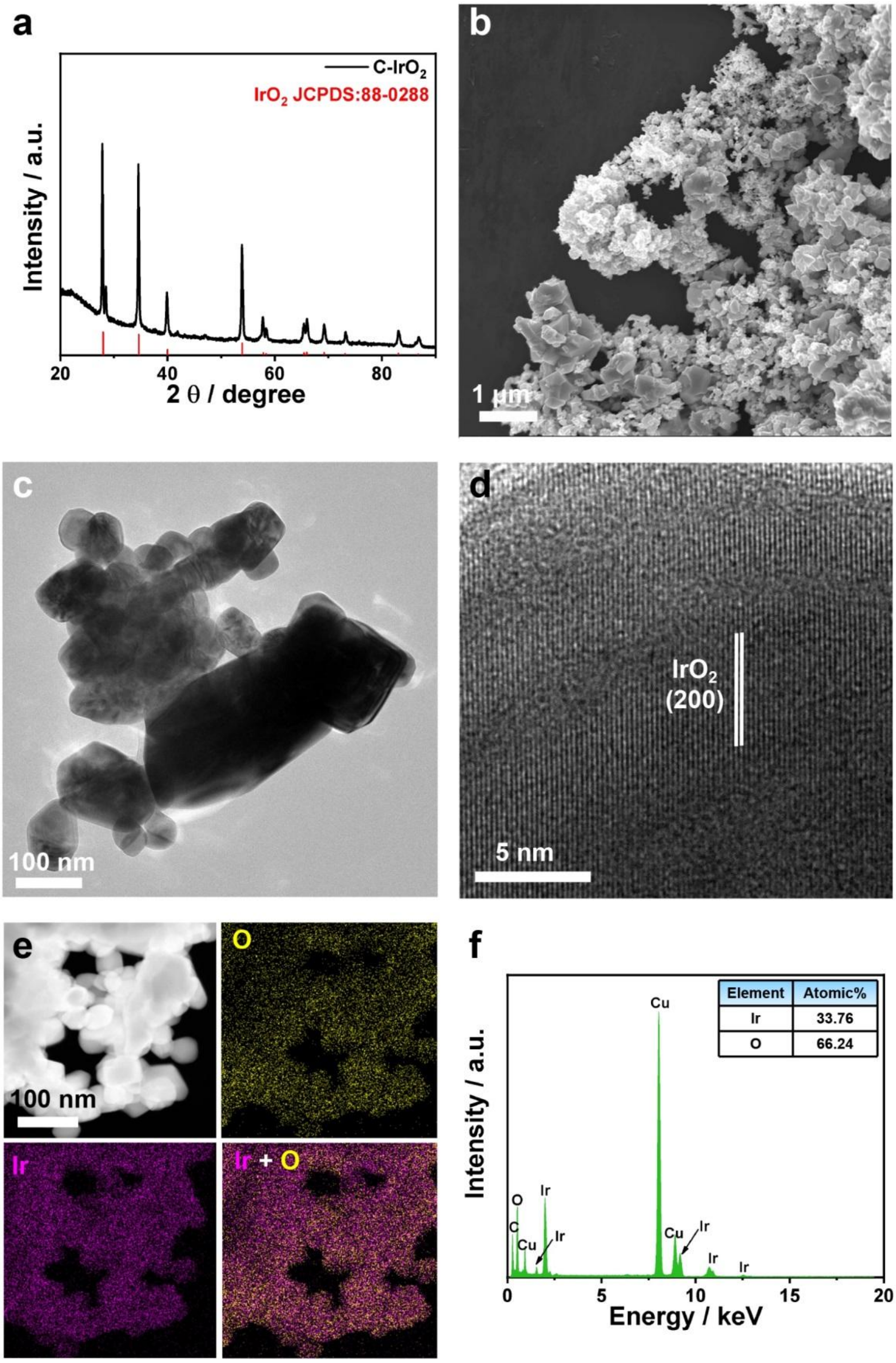

Supplementary Fig. 5 | Characterizations of C-IrO_2. a, XRD pattern of C-IrO_2. **b,** SEM, **c,** TEM and **d,** HRTEM images of C-IrO_2. **e,** STEM-EDX mapping of C-IrO_2. **f,** TEM-EDX spectrum of C-IrO_2, and the table shows the atomic ratio of Ir to O in C-IrO_2.

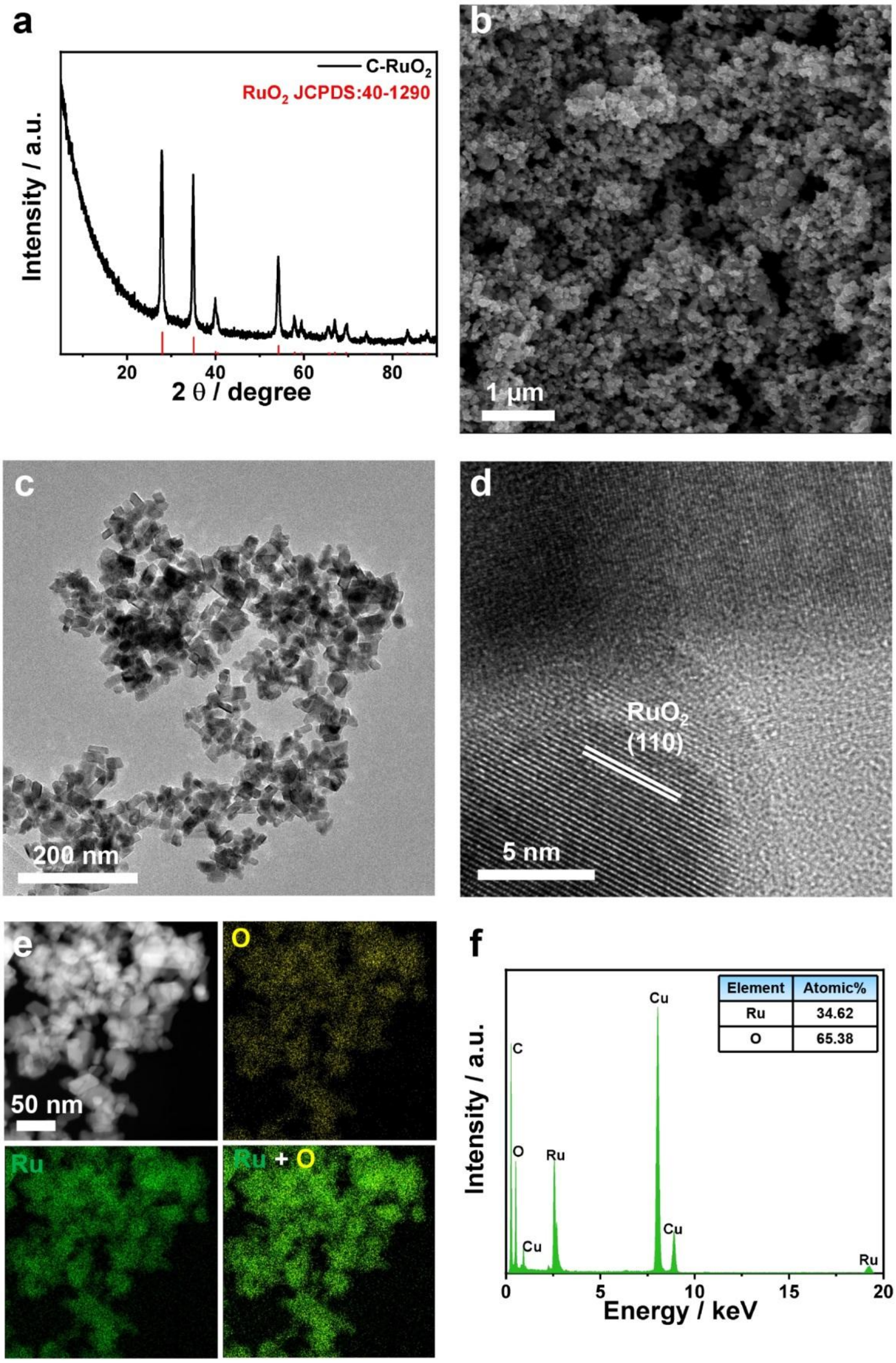

Supplementary Fig. 6 | Characterizations of C-RuO$_2$. a, XRD pattern of C-RuO$_2$. **b,** SEM, **c,** TEM and **d,** HRTEM images of C-RuO$_2$. **e,** STEM-EDX mapping of C-RuO$_2$. **f,** TEM-EDX spectrum of C-RuO$_2$, and the table shows the atomic ratio of Ru to O in C-RuO$_2$.

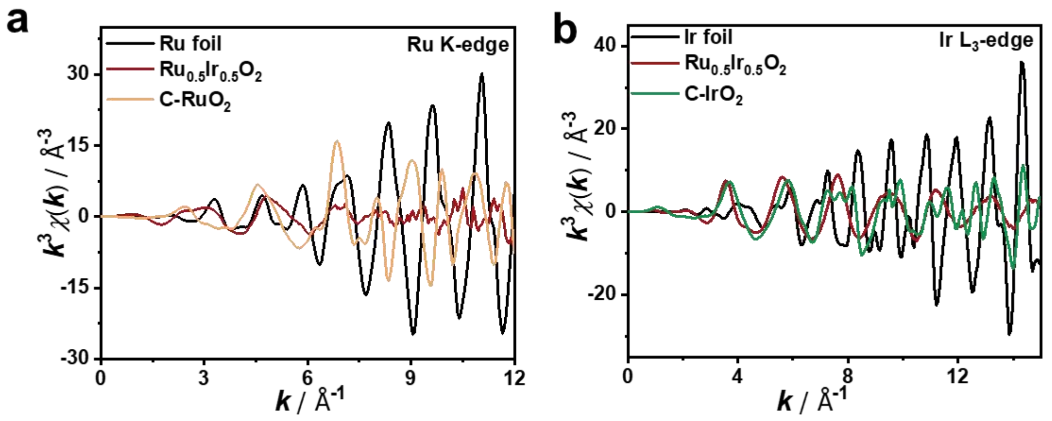

Supplementary Fig. 7 | EXAFS oscillations of $Ru_{0.5}Ir_{0.5}O_2$ electrocatalyst and the reference samples. **a,** Ru K-edge EXAFS oscillations of $Ru_{0.5}Ir_{0.5}O_2$, C-RuO_2 and Ru foil reference. **b,** Ir L_3-edge EXAFS oscillations of $Ru_{0.5}Ir_{0.5}O_2$, C-IrO_2 and Ir foil reference.

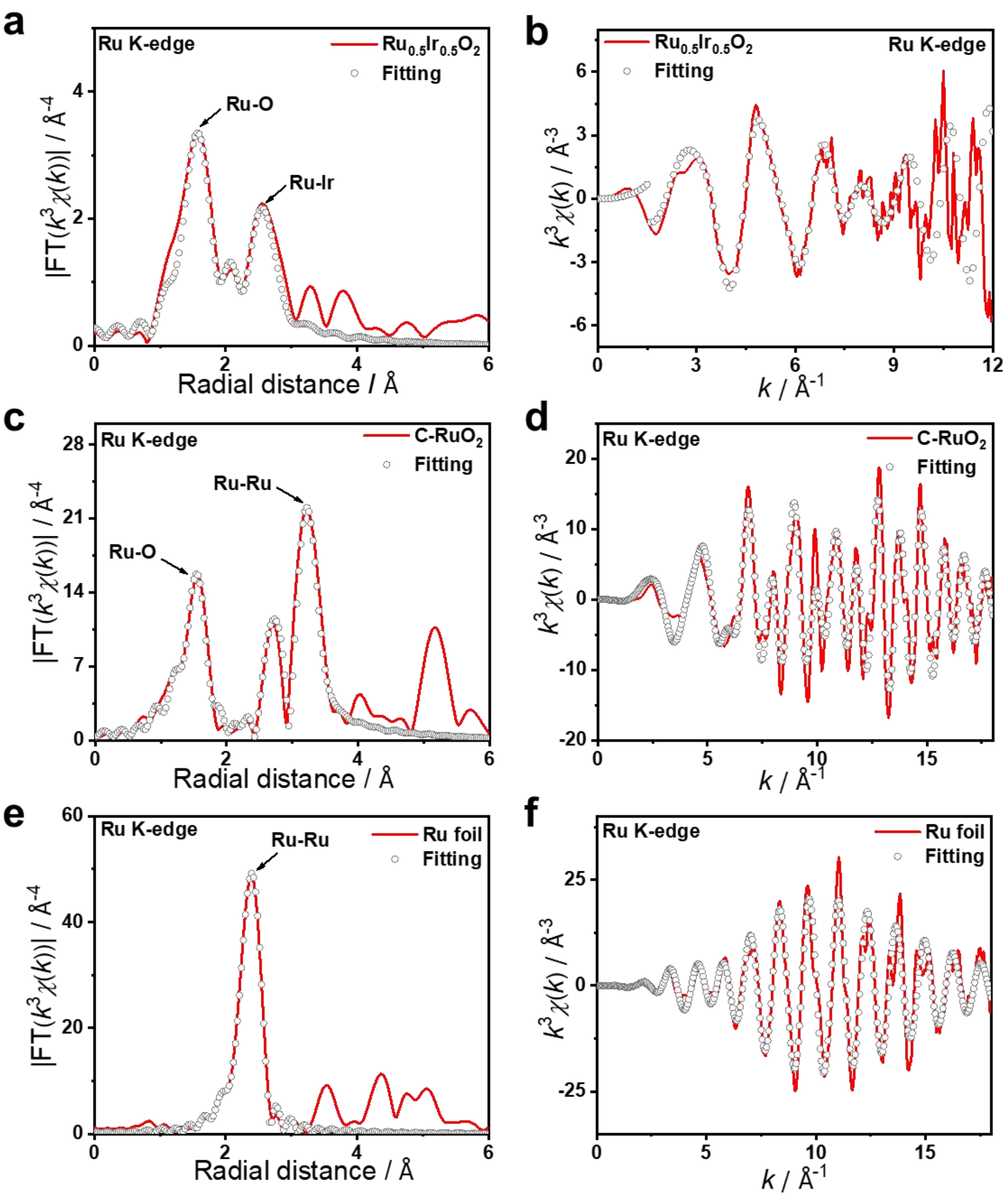

Supplementary Fig. 8 | R space and inverse FT-EXAFS fitting of $Ru_{0.5}Ir_{0.5}O_2$ electrocatalyst and the reference samples. R space and inverse FT-EXAFS fitting result of Ru K-edge for (**a** and **b**) $Ru_{0.5}Ir_{0.5}O_2$, (**c** and **d**) C-RuO_2 and (**e** and **f**) Ru foil reference.

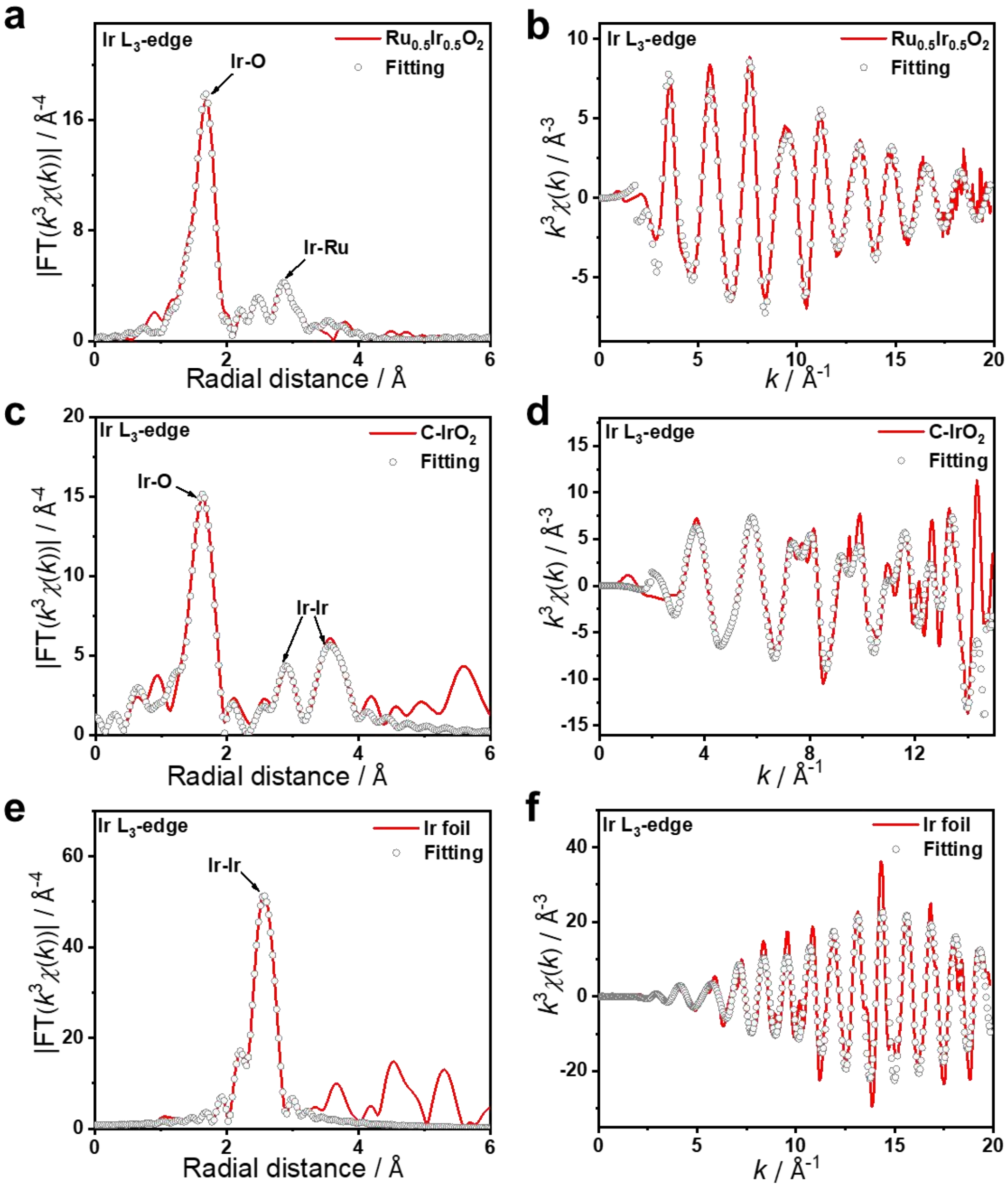

Supplementary Fig. 9 | R space and inverse FT-EXAFS fitting of $Ru_{0.5}Ir_{0.5}O_2$ electrocatalyst and the reference samples. R space and inverse FT-EXAFS fitting result of Ir L_3-edge for (**a** and **b**) $Ru_{0.5}Ir_{0.5}O_2$, (**c** and **d**) C-IrO_2 and (**e** and **f**) Ir foil reference.

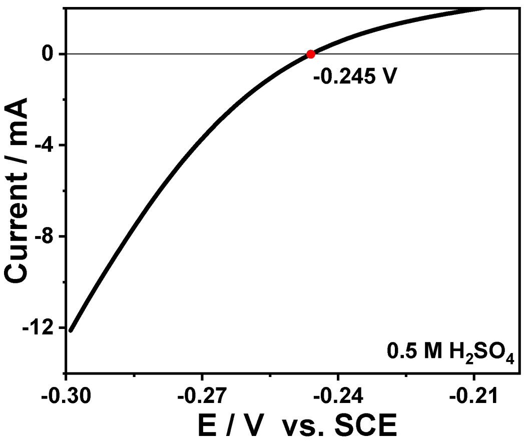

Supplementary Fig. 10 | Calibration of the saturated calomel electrode (SCE). The SCE electrode with respect to reversible hydrogen electrode (RHE) in 0.5 M H_2SO_4 electrolyte bubbled with pure hydrogen gas at room temperature. Scan rate: 5 mV s^{-1}. The average of the two potentials of the current crossing zero is considered the thermodynamic potential of the hydrogen electrode reaction. In 0.5 M H_2SO_4 electrolyte, E (vs. RHE) = E (vs. SCE) + 0.245 V.

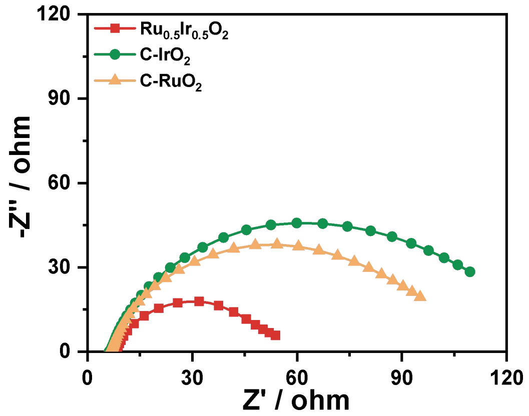

Supplementary Fig. 11 | EIS measurements of different catalysts. Nyquist plot of $Ru_{0.5}Ir_{0.5}O_2$ in O_2-saturated 0.5 M H_2SO_4 electrolyte (7.5 ± 0.1 ohms), Nyquist plot of C-IrO_2 in O_2-saturated 0.5 M H_2SO_4 electrolyte (6.2 ± 0.2 ohms) and Nyquist plot of C-RuO_2 in O_2-saturated 0.5 M H_2SO_4 electrolyte (6.3 ± 0.2 ohms).

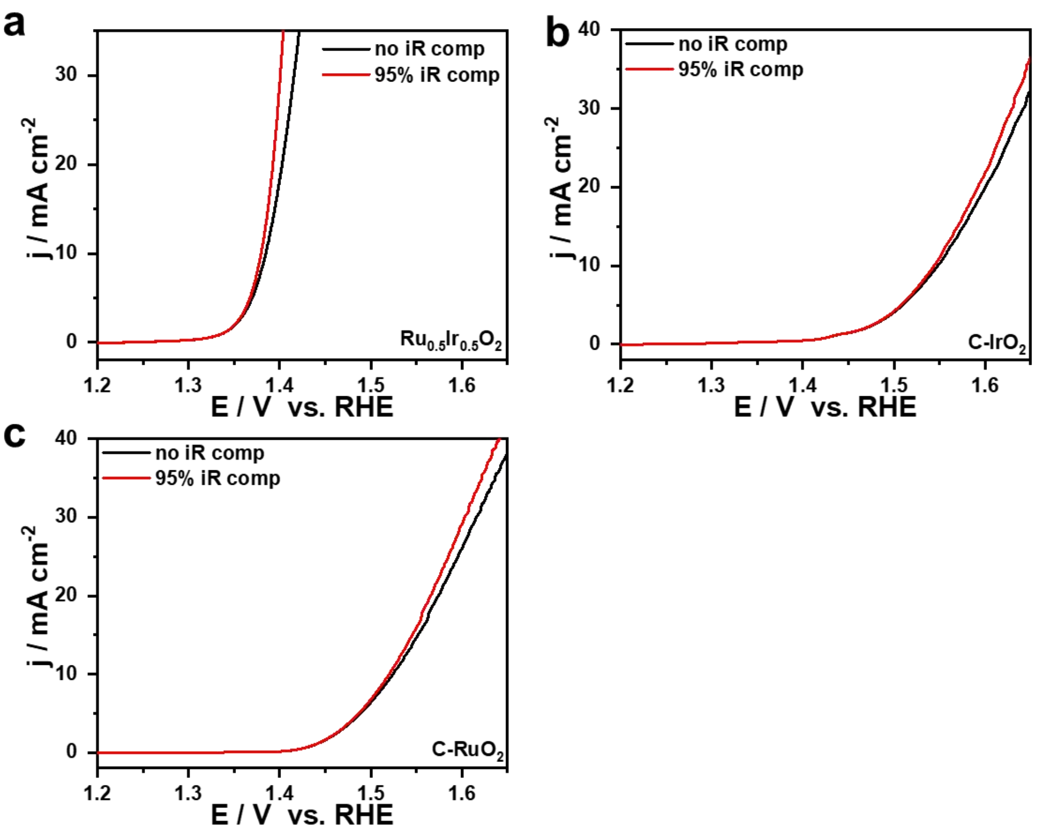

Supplementary Fig. 12 | Polarization curves with 95% *iR* compensation. a, Polarization curves of $Ru_{0.5}Ir_{0.5}O_2$ under 95% *iR* compensation levels on GCE (3 mm in diameter) in O_2-saturated 0.5 M H_2SO_4 electrolyte (7.5 ± 0.1 ohms). **b,** Polarization curves of C-IrO_2 under 95% *iR* compensation levels on GCE (3 mm in diameter) in O_2-saturated 0.5 M H_2SO_4 electrolyte (6.2 ± 0.2 ohms). **c,** Polarization curves of C-RuO_2 under 95% *iR* compensation levels on GCE (3 mm in diameter) in O_2-saturated 0.5 M H_2SO_4 electrolyte (6.3 ± 0.2 ohms).

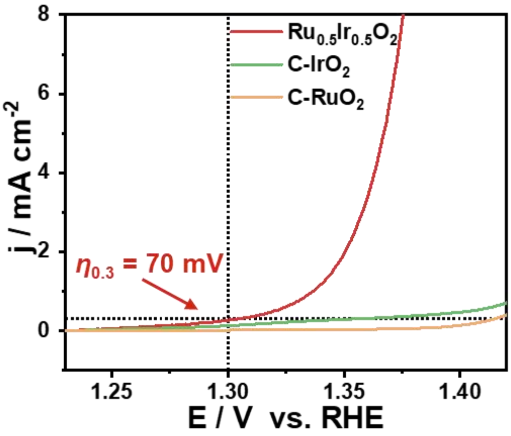

Supplementary Fig. 13 | Polarization curves. LSV curves of $Ru_{0.5}Ir_{0.5}O_2$, C-IrO_2 and C-RuO_2 in O_2-saturated 0.5 M H_2SO_4 electrolyte with *iR*-correction.

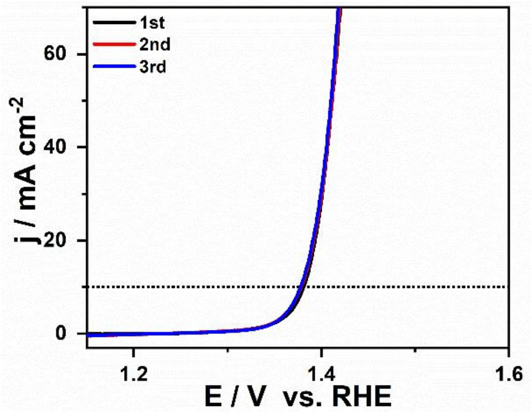

Supplementary Fig. 14 | Reproducibility of linear sweep voltammetry (LSV) scans for $Ru_{0.5}Ir_{0.5}O_2$ catalyst. The OER polarization curves of $Ru_{0.5}Ir_{0.5}O_2$ in O_2-saturated 0.5 M H_2SO_4 electrolyte. The synthetic $Ru_{0.5}Ir_{0.5}O_2$ was dispersed 5 mg in 0.9 mL isopropanol solution and 0.1 mL Nafion solution (0.5 wt%), and the ink was formed by ultrasonically ultrasonic action for 1 h to form a homogeneous ink. Then 4 μL of suspension was loaded on glass carbon electrode (GCE, 3 mm in diameter) to prepared the working electrode (mass loading ~ 283 μg cm^{-2}). Finally, the prepared working electrode was dried in room temperature. Scan rate: 5 mV s^{-1}.

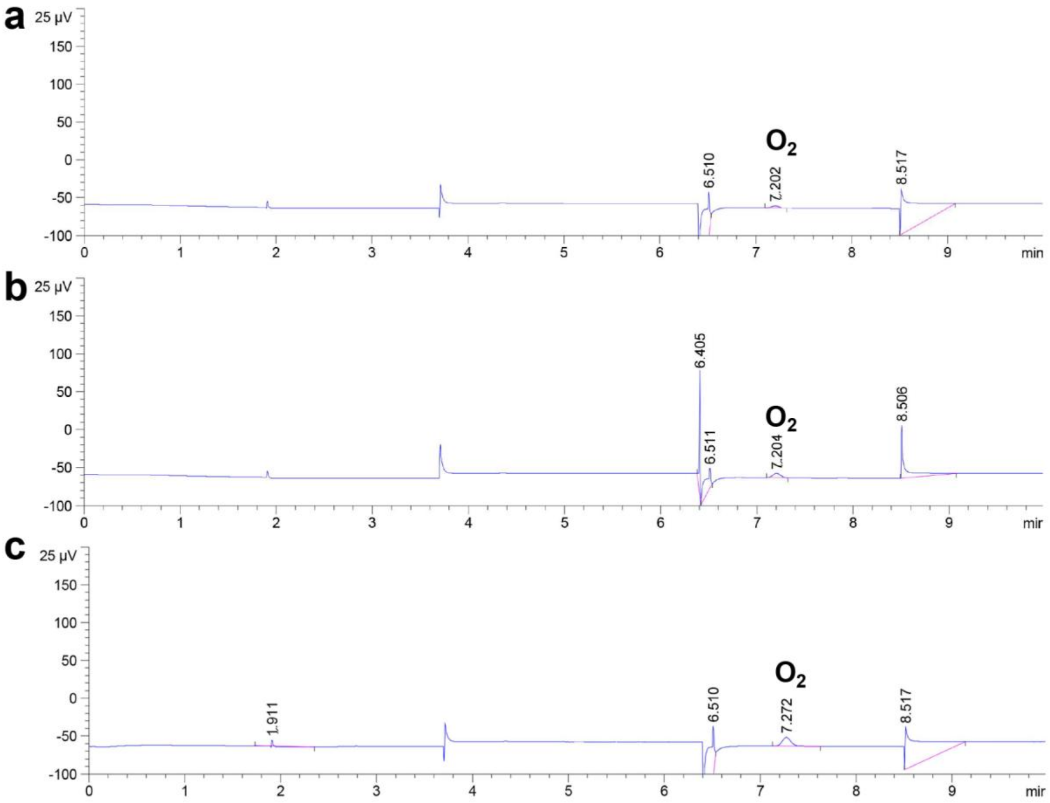

Supplementary Fig. 15 | Gas chromatographic (GC) analysis of $Ru_{0.5}Ir_{0.5}O_2$. GC data of the produced oxygen by $Ru_{0.5}Ir_{0.5}O_2$ at **a,** 20 mA cm^{-2}, **b,** 40 mA cm^{-2}, **c,** 100 mA cm^{-2} in 0.5 M H_2SO_4, respectively.

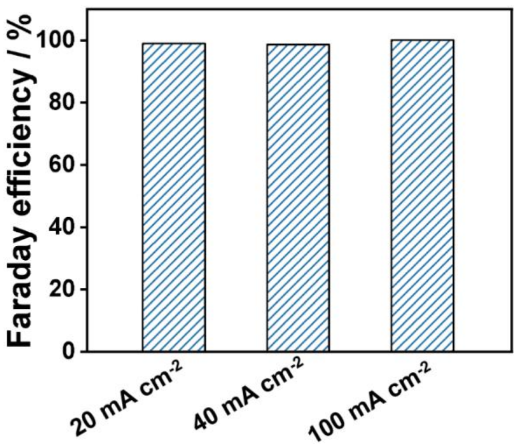

Supplementary Fig. 16 | O_2 Faraday efficiencies of $Ru_{0.5}Ir_{0.5}O_2$ via using the gas chromatography analyses technique. O_2 Faraday efficiencies of $Ru_{0.5}Ir_{0.5}O_2$ at 20 mA cm^{-2}, 40 mA cm^{-2} and 100 mA cm^{-2}.

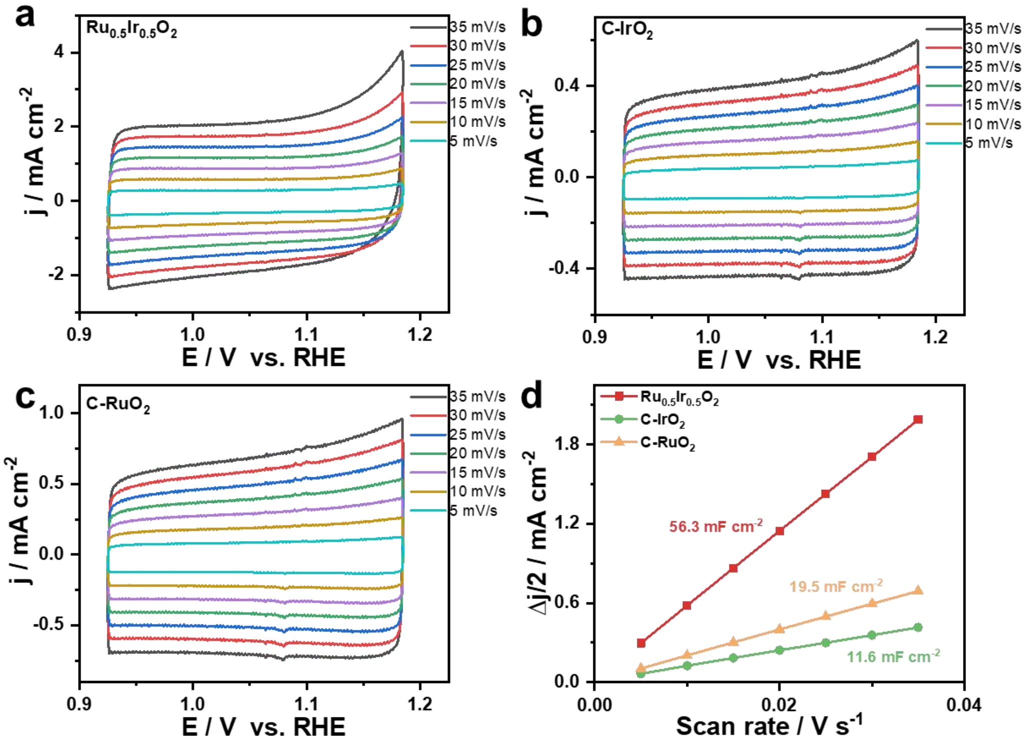

Supplementary Fig. 17 | ECSA characterizations of catalysts. CV curves of (**a**) $Ru_{0.5}Ir_{0.5}O_2$, (**b**) C-IrO_2 and (**c**) C-RuO_2 catalysts in the non-Faradaic region with the scan rates of 5, 10, 15, 20, 25, 30, and 35 mV s^{-1} in 0.5 M H_2SO_4 electrolyte. **d**, C_{dl} plots obtained from CV curves.

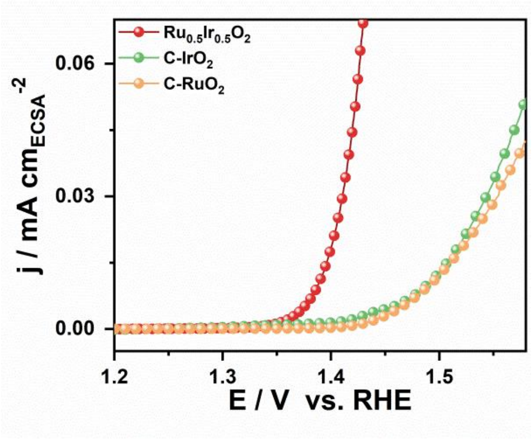

Supplementary Fig. 18 | OER performance. Normalized LSV curves to electrochemically active surface area (ECSA) of $Ru_{0.5}Ir_{0.5}O_2$, C-IrO_2 and C-RuO_2 catalysts.

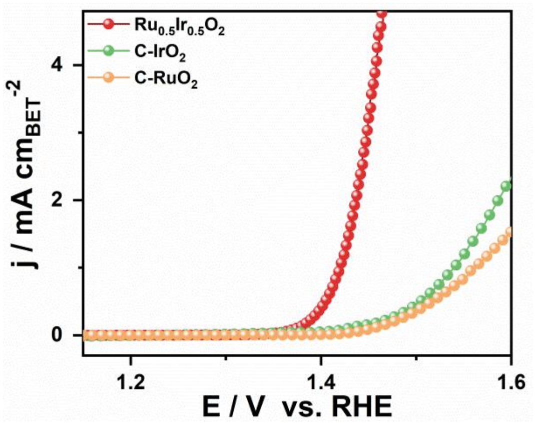

Supplementary Fig. 19 | OER performance. Normalized LSV curves to BET-based areas of $Ru_{0.5}Ir_{0.5}O_2$, C-IrO_2 and C-RuO_2.

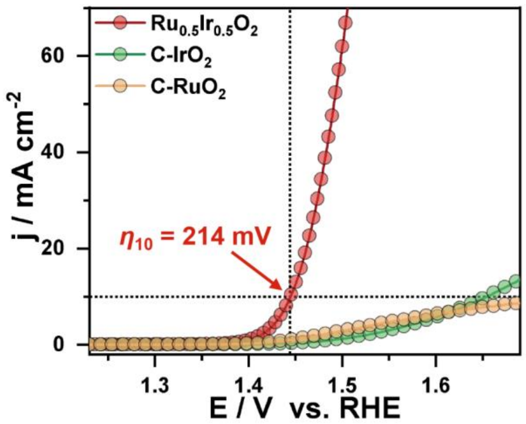

Supplementary Fig. 20 | Polarization curves of catalysts after stability test. LSV curves of $Ru_{0.5}Ir_{0.5}O_2$, C-IrO_2 and C-RuO_2 in O_2-saturated 0.5 M H_2SO_4 electrolyte with *iR*-correction after a 618.3 h stability test at 10 mA cm^{-2}, where $Ru_{0.5}Ir_{0.5}O_2$ deliver an overpotential of 214 mV to achieve the current density of 10 mA cm^{-2}.

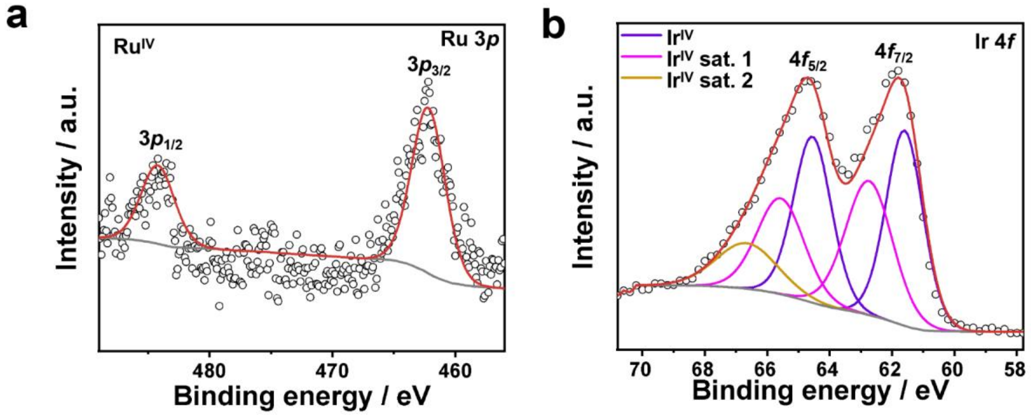

Supplementary Fig. 21 | XPS characterizations of $Ru_{0.5}Ir_{0.5}O_2$ after long-term stability test at 10 mA cm^{-2}. a, Ru 3*p* and **b,** Ir 4*f* XPS spectra of $Ru_{0.5}Ir_{0.5}O_2$ after the stability test.

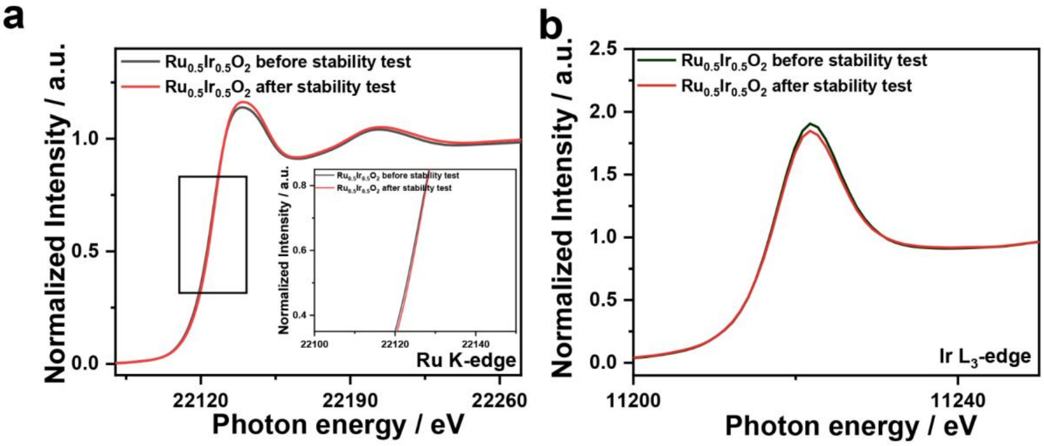

Supplementary Fig. 22 | XAS characterizations of $Ru_{0.5}Ir_{0.5}O_2$ catalyst after the long term stability test. a, Ru XANES spectra at the Ru K-edge of $Ru_{0.5}Ir_{0.5}O_2$ before and after the stability test. **b,** Ir XANES spectra at Ir L_3-edge of $Ru_{0.5}Ir_{0.5}O_2$ before and after the stability test.

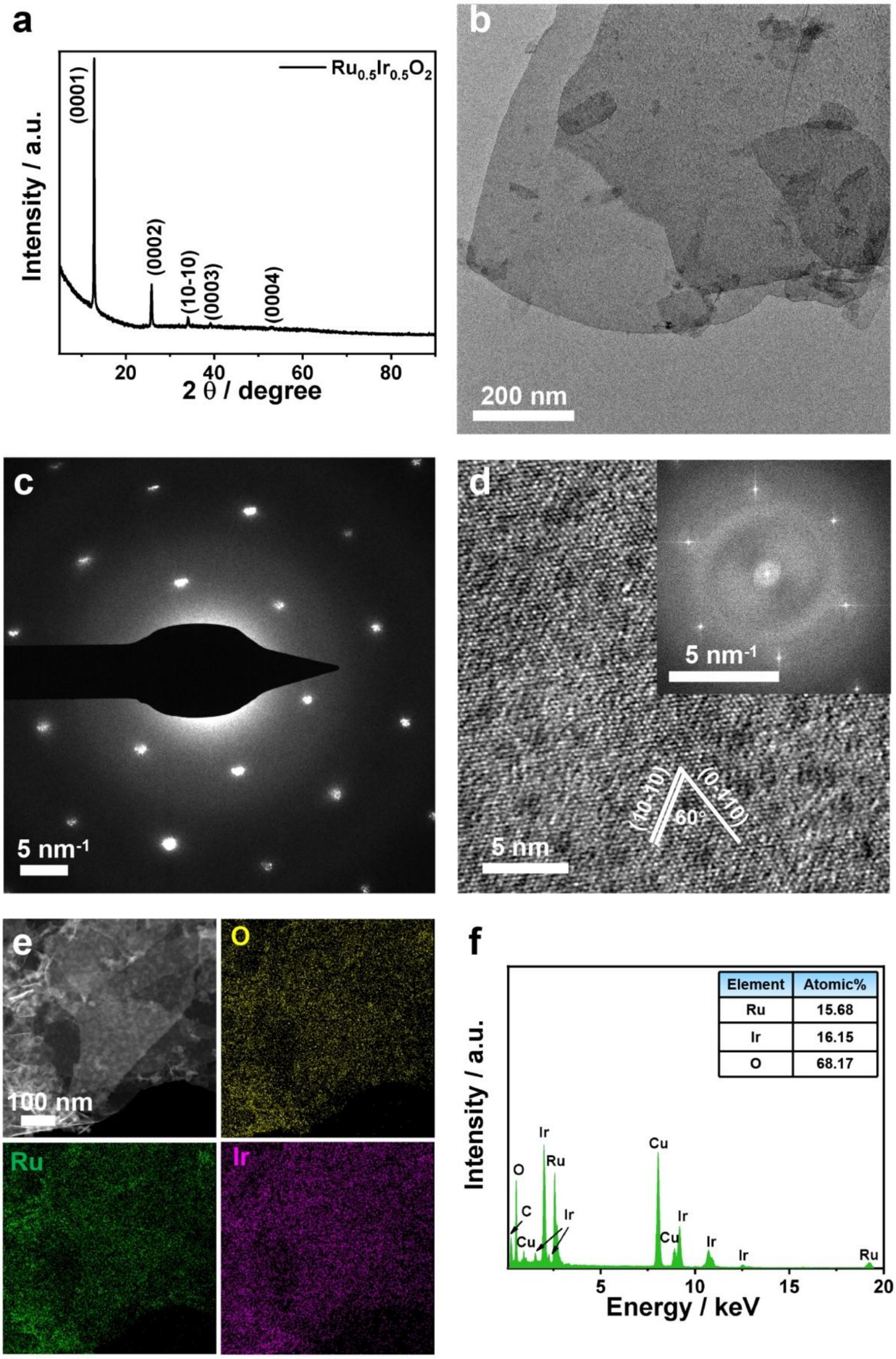

Supplementary Fig. 23 | Characterizations of $Ru_{0.5}Ir_{0.5}O_2$ after long-term stability test at 10 mA cm^{-2}. a, XRD pattern of $Ru_{0.5}Ir_{0.5}O_2$ after the stability test. **b,** TEM and **c,** SAED pattern of $Ru_{0.5}Ir_{0.5}O_2$ after the stability test. **d,** HRTEM images of $Ru_{0.5}Ir_{0.5}O_2$ after the stability test. The inset in (**d**) shows the FFT of the area. **e,** STEM-EDX mapping of $Ru_{0.5}Ir_{0.5}O_2$ after stability test. **f,** TEM-EDX spectrum of $Ru_{0.5}Ir_{0.5}O_2$ after stability test.

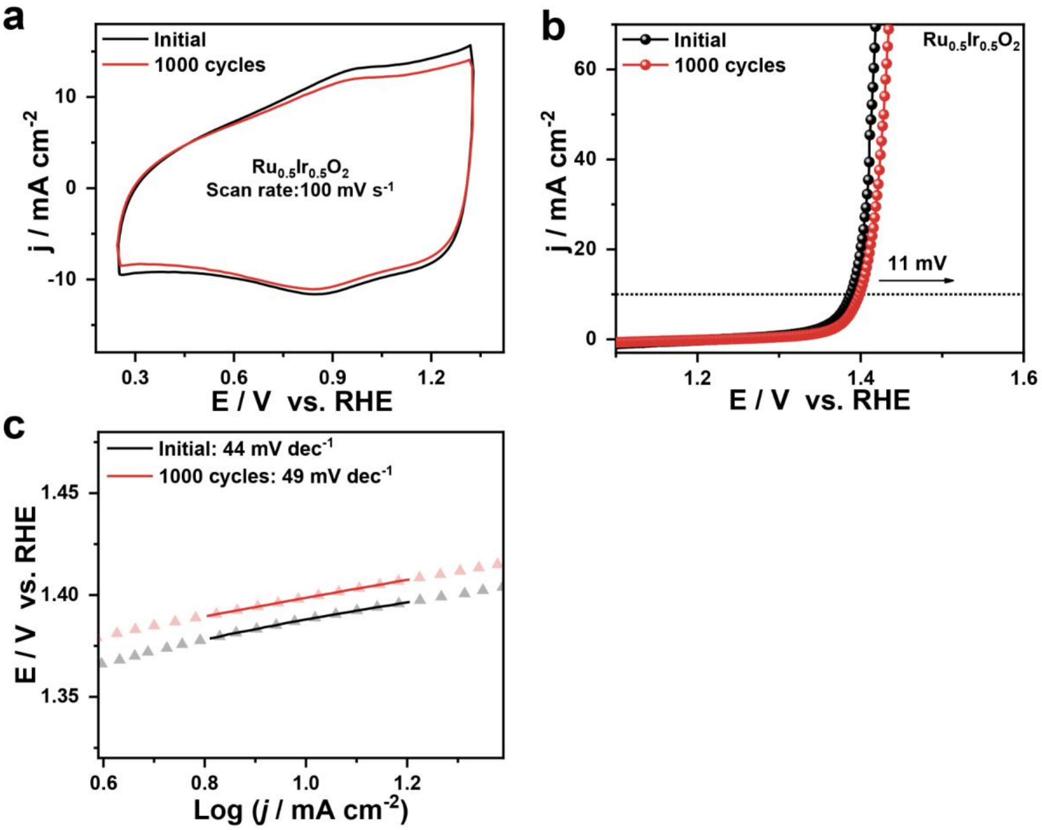

Supplementary Fig. 24 | ADT-CV test of $Ru_{0.5}Ir_{0.5}O_2$. a, Cyclic voltammogram curves of $Ru_{0.5}Ir_{0.5}O_2$ in a potential region of 0.25 ~ 1.3 V vs. RHE with the scan rate of 100 mV s^{-1}. **b,** The OER polarization curves of $Ru_{0.5}Ir_{0.5}O_2$ before and after 1000 CV cycles. **c,** The Tafel slopes of $Ru_{0.5}Ir_{0.5}O_2$ before and after the ADT-CV testing.

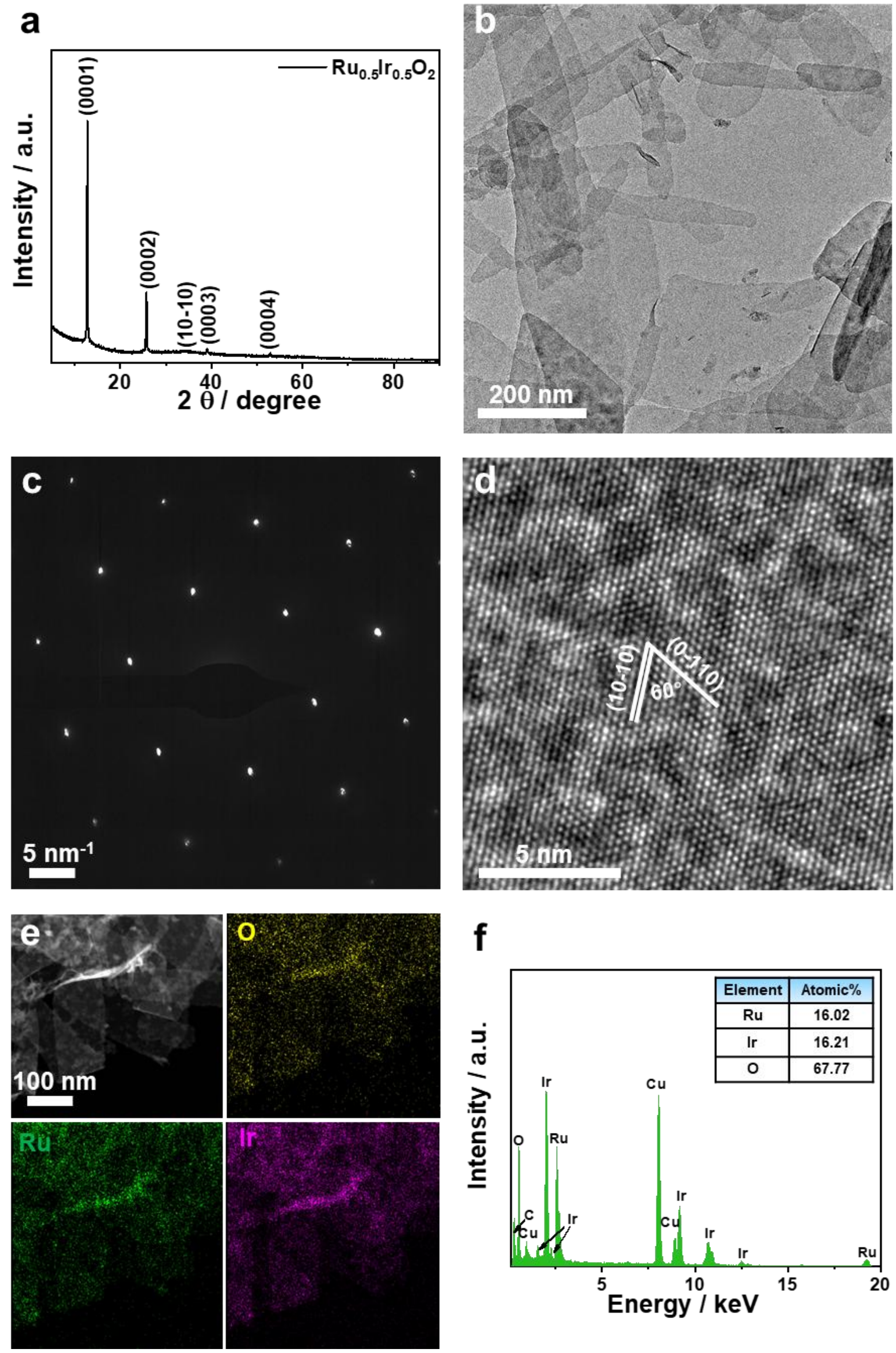

Supplementary Fig. 25 | Characterizations of $Ru_{0.5}Ir_{0.5}O_2$ after ADT-CV testing. a, XRD pattern of $Ru_{0.5}Ir_{0.5}O_2$ after ADT-CV testing. **b,** TEM image of $Ru_{0.5}Ir_{0.5}O_2$, **c**, the SAED pattern and **d,** HRTEM image of $Ru_{0.5}Ir_{0.5}O_2$ after the ADT-CV testing. **e,** STEM-EDX mapping of $Ru_{0.5}Ir_{0.5}O_2$ and **f**, TEM-EDX spectrum of $Ru_{0.5}Ir_{0.5}O_2$ after ADT-CV testing.

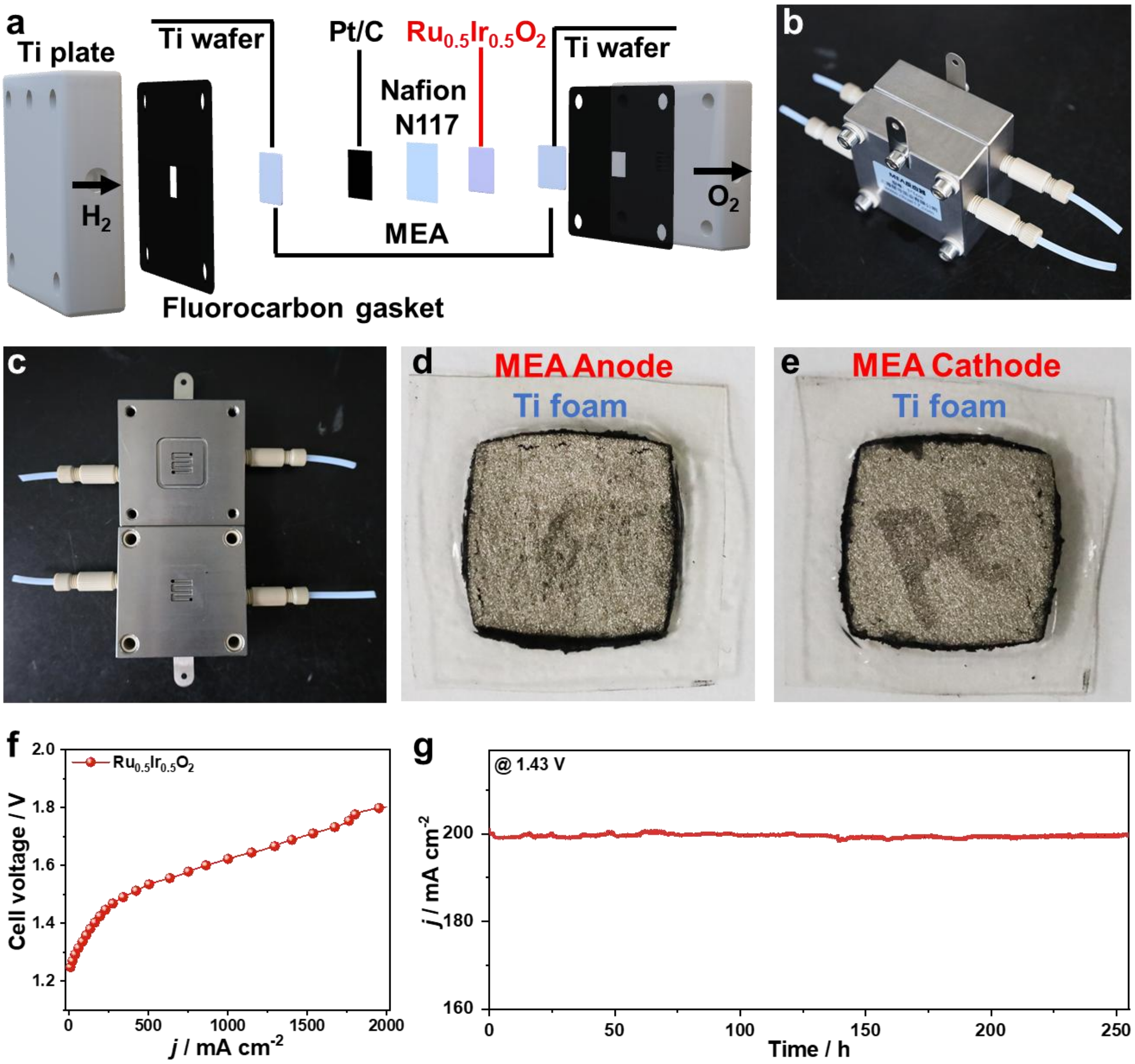

Supplementary Fig. 26 | Electrocatalytic properties of $Ru_{0.5}Ir_{0.5}O_2$ in 0.5 M H_2SO_4 electrolyte in PEM electrolyser. **a**, Schematic illustration of the PEM device. **b,** The top-view and **c,** side-view optical images of the assembled PEM electrolyser. **d**, The optical image of the anode side of the MEA electrode with Ti wafer as the gas diffusion layer. **e,** The optical image of the cathode side of the MEA electrode with Ti wafer as the gas diffusion layer. **f,** The polarization curve of $Ru_{0.5}Ir_{0.5}O_2$ in the PEM electrolyser. **g,** Chronoamperometry stability test of $Ru_{0.5}Ir_{0.5}O_2$ catalyst (with a mass loading of 1.0 mg cm^{-2}) at the cell voltage of 1.43 V in acidic PEM electrolyser at room temperature.

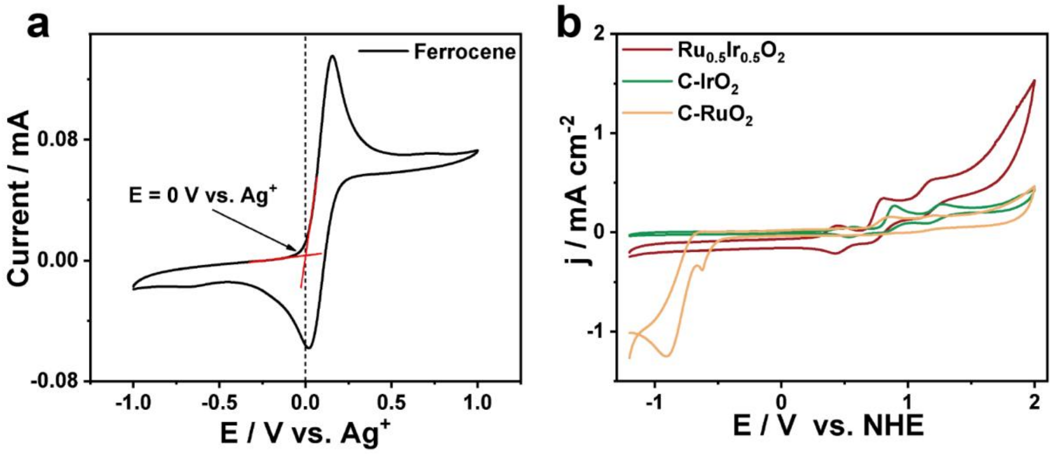

Supplementary Fig. 27 | Cyclic voltammetry (CV) test in anhydrous acetonitrile. a, The CV of ferrocene in anhydrous acetonitrile with the scan rate of 30 mV s^{-1}. E (NHE) = E(Ag^+) + 0.4 V. **b**, The CV curves of $Ru_{0.5}Ir_{0.5}O_2$, C-IrO_2 and C-RuO_2 in anhydrous acetonitrile with the scan rate of 30 mV s^{-1}.

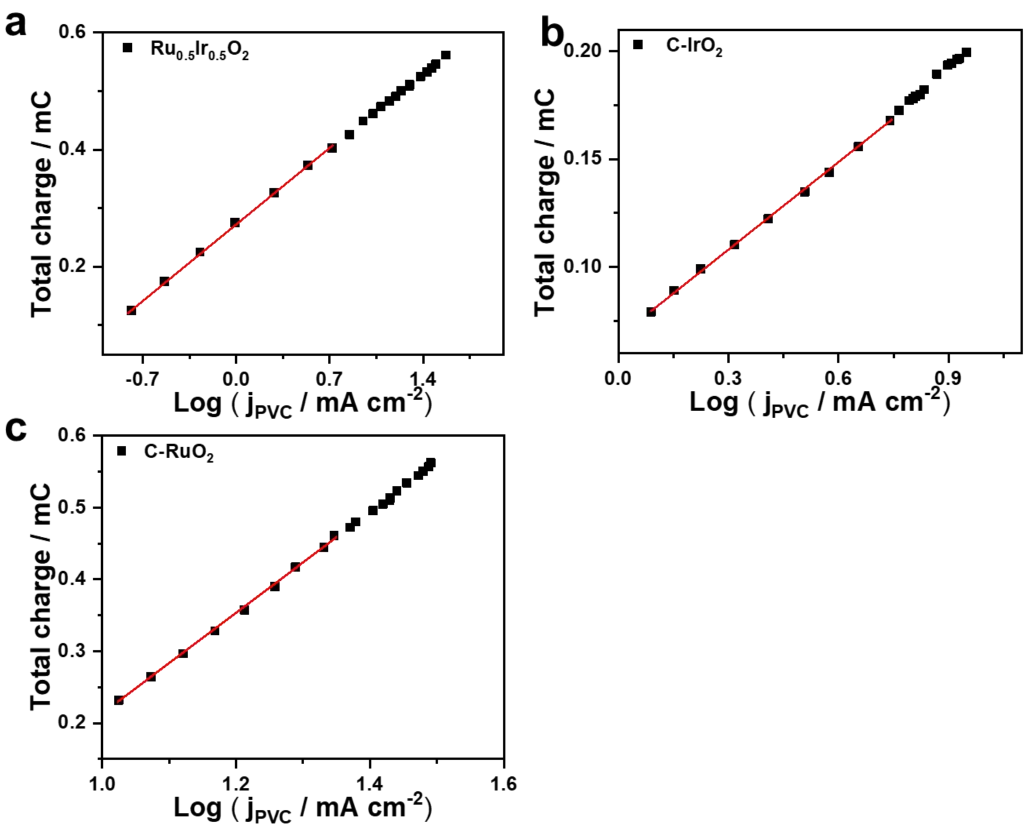

Supplementary Fig. 28 | Total charge (integral anodic charge) vs. log OER current densities of $Ru_{0.5}Ir_{0.5}O_2$ (**a**), C-IrO_2 (**b**) and C-RuO_2 (**c**) from PVC measurements. The total charge data is derived from the charge integral of the PVC test, and the current density data is derived from the OER current in the PVC test.

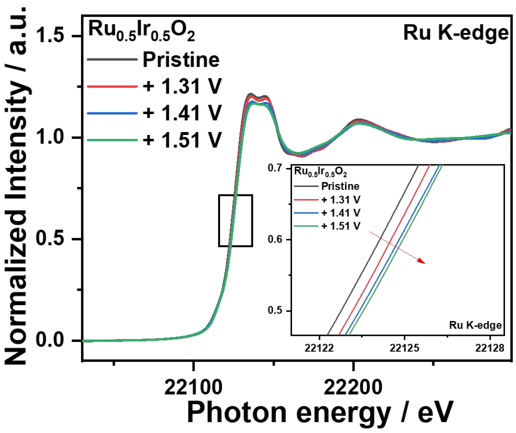

Supplementary Fig. 29 | In situ XAS characterization of $Ru_{0.5}Ir_{0.5}O_2$. In situ Ru K edge XANES spectra of $Ru_{0.5}Ir_{0.5}O_2$ with applied bias rise from 1.31 V to 1.51 V vs. RHE.

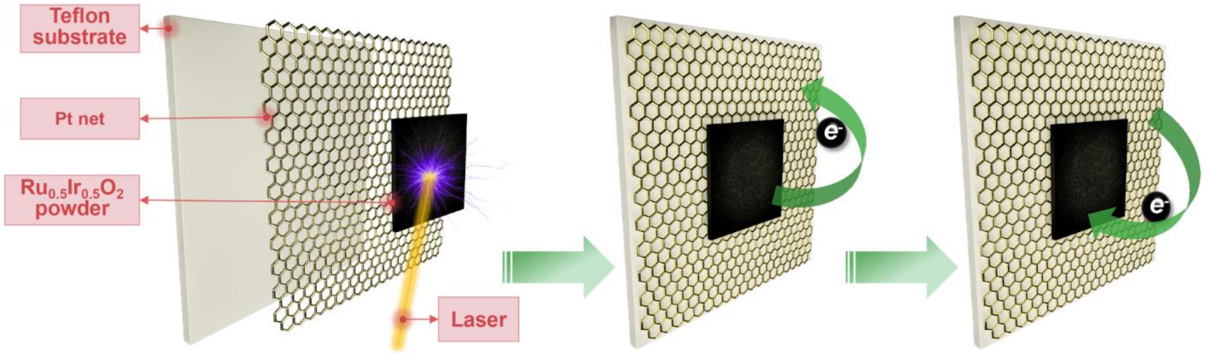

Supplementary Fig. 30 | Transient photo-induced voltage (TPV) measurements. Schematic diagram of TPV test device.

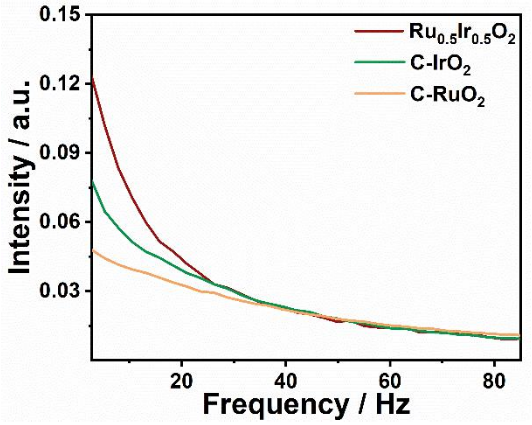

Supplementary Fig. 31 | Transformation of TPV data. FFT curves of $Ru_{0.5}Ir_{0.5}O_2$, C-IrO_2 and C-RuO_2.

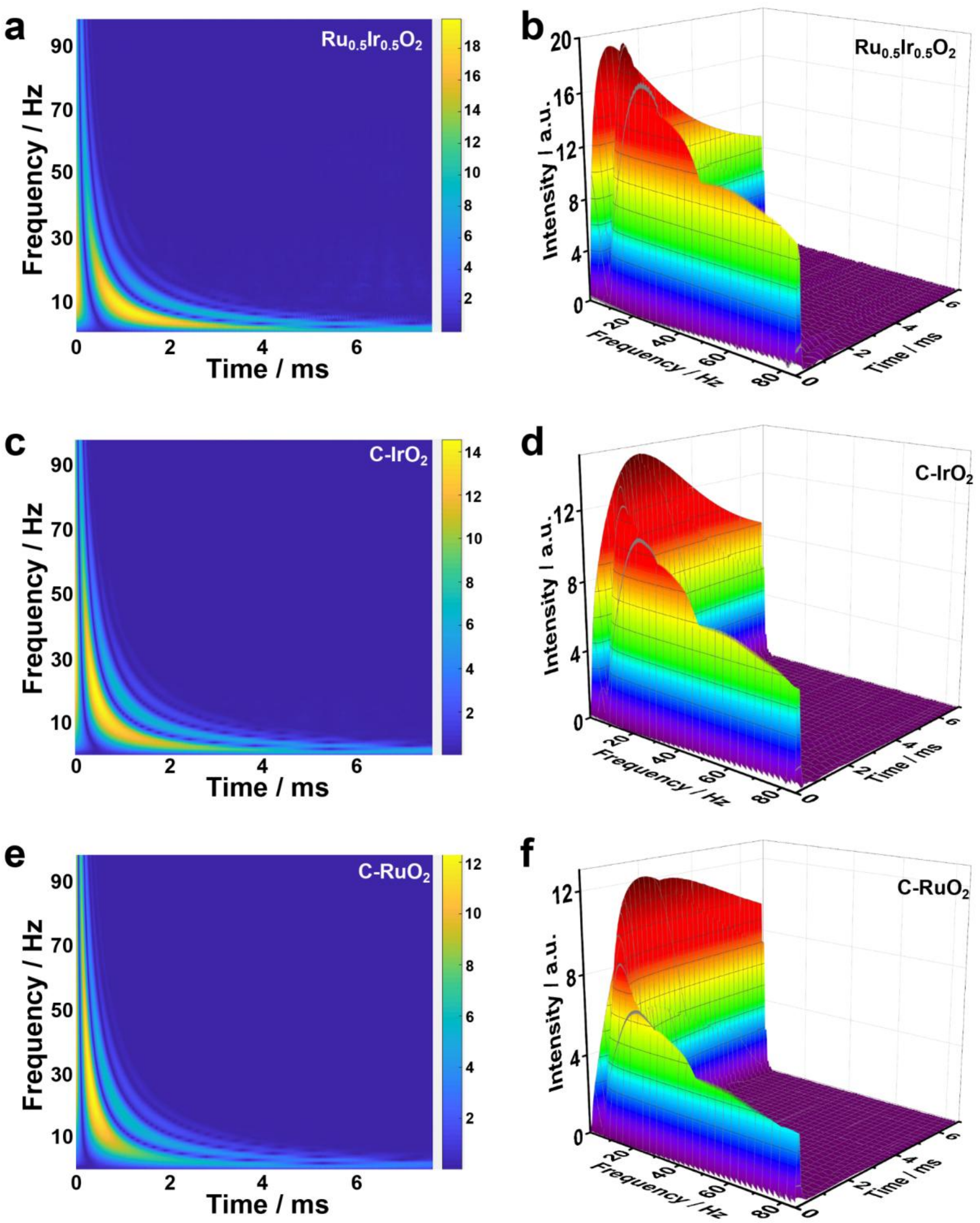

Supplementary Fig. 32 | Transformation of TPV data. **a**, **c**, and **e**, 2D CWT patterns and **b**, **d**, and **f**, 3D CWT spectrum of $Ru_{0.5}Ir_{0.5}O_2$, C-IrO_2 and C-RuO_2, respectively.

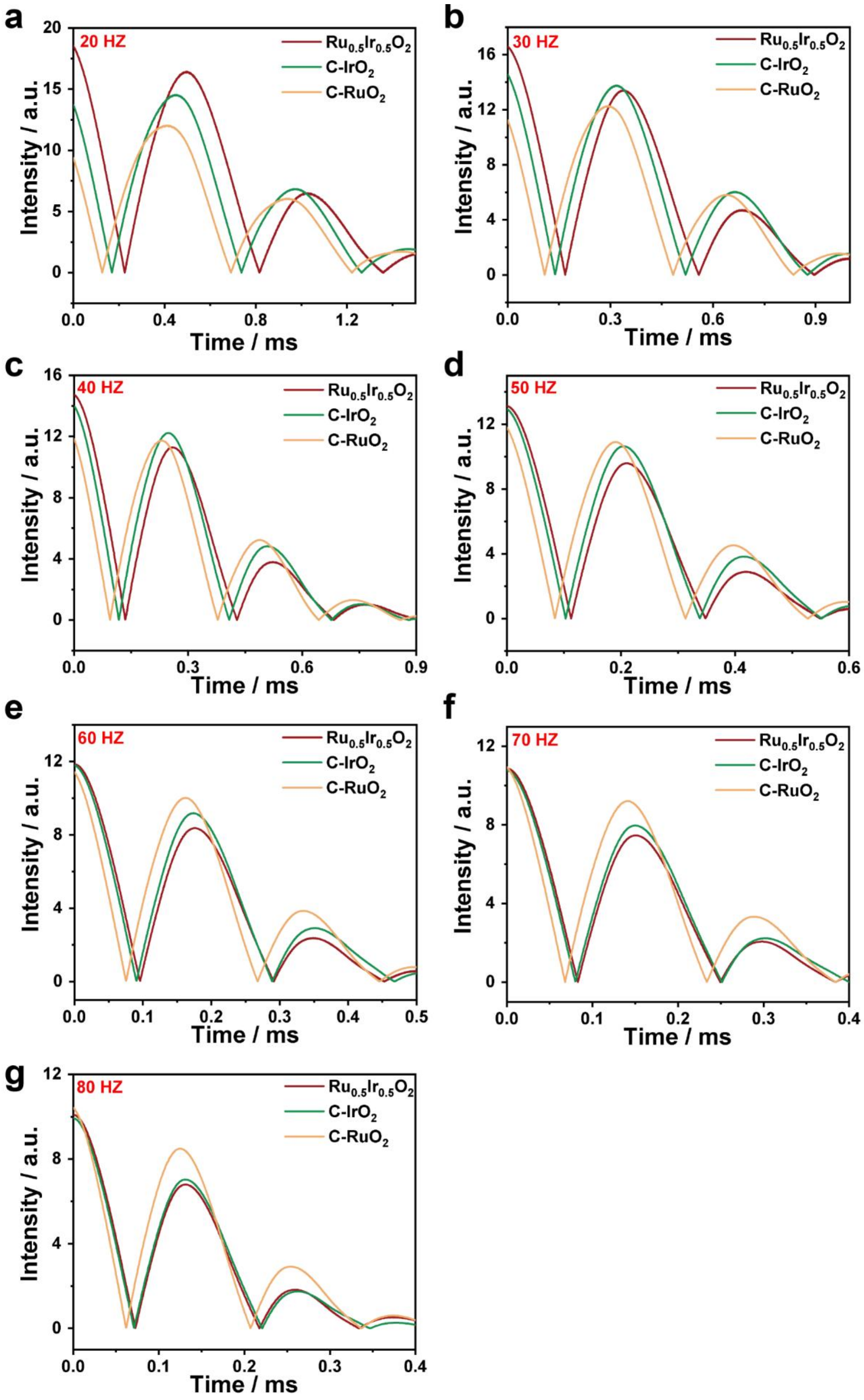

Supplementary Fig. 33 | Relationships between intensity and time of peak positions at different frequencies. a-g, Intensity-Time curves of $Ru_{0.5}Ir_{0.5}O_2$ at different frequencies (20-80 Hz).

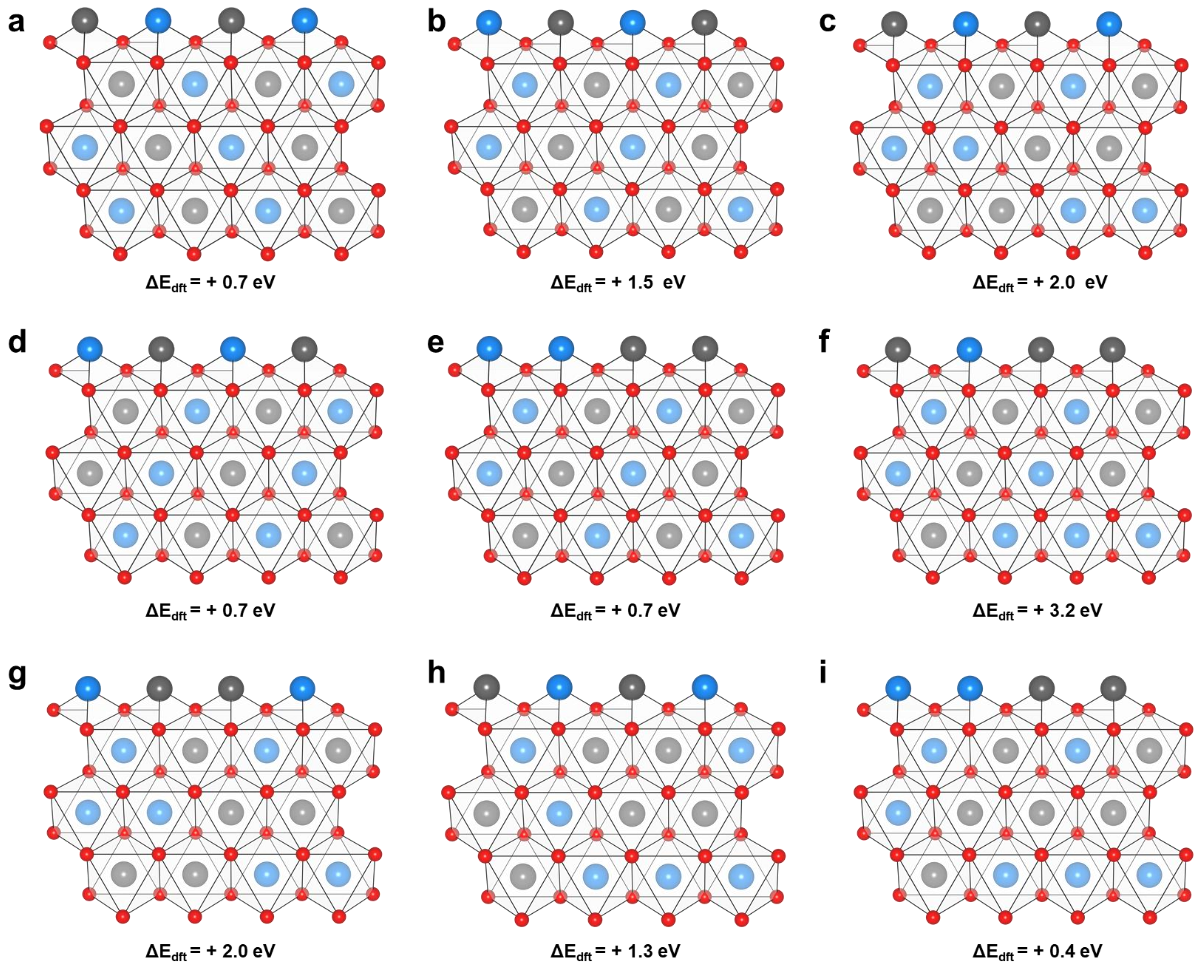

Supplementary Fig. 34 | Calculation of $Ru_{0.5}Ir_{0.5}O_2$ structural stability. (**a**) to (**i**) represent the structures and ΔE_{dft} of $Ru_{0.5}Ir_{0.5}O_2$ with different structure types and all the potential energies are referenced to the most stable structure (-331.4 eV) in DFT calculations. The Ir, Ru and O atoms are represented with the grey, blue and red, respectively. In our DFT models for $Ru_{0.5}Ir_{0.5}O_2$, we approached the problem by expanding the 2D IrO_2 which is obtained from XRD by 4 times along the x and y direction, and substituting Ir atoms with Ru atoms to reach an atomic ratio of Ru/Ir = 1:1, which aligns closely with the experimental value, we calculated some different structures $Ru_{0.5}Ir_{0.5}O_2$ (01-10) surface, and pick the most stable structure as our calculated model.

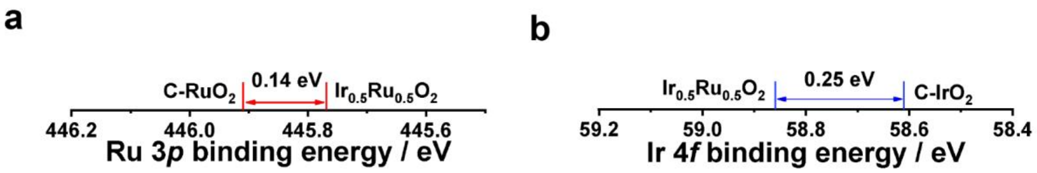

Supplementary Fig. 35 | The XPS simulation. a, Ru 3*p* XPS simulation spectrum of $Ru_{0.5}Ir_{0.5}O_2$ and C-RuO_2. **b**, Ir 4*f* XPS simulation spectrum of $Ru_{0.5}Ir_{0.5}O_2$ and C-IrO_2

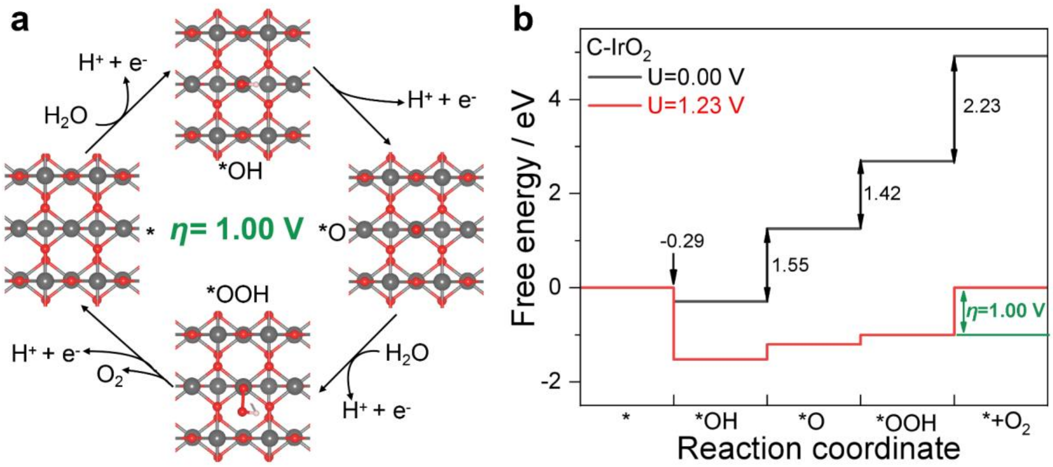

Supplementary Fig. 36 | Mechanism of OER steps and the free energy profile of OER over the C-IrO$_2$ catalyst. a, Schematic illustration of OER mechanism on the C-IrO$_2$ (grey, Ir; red, O; white, H). **b**, The reaction paths on C-IrO$_2$ catalyst with the set potential of 0 and 1.23 V. The η value indicates the rate of the catalyst determining the overpotential of the step.

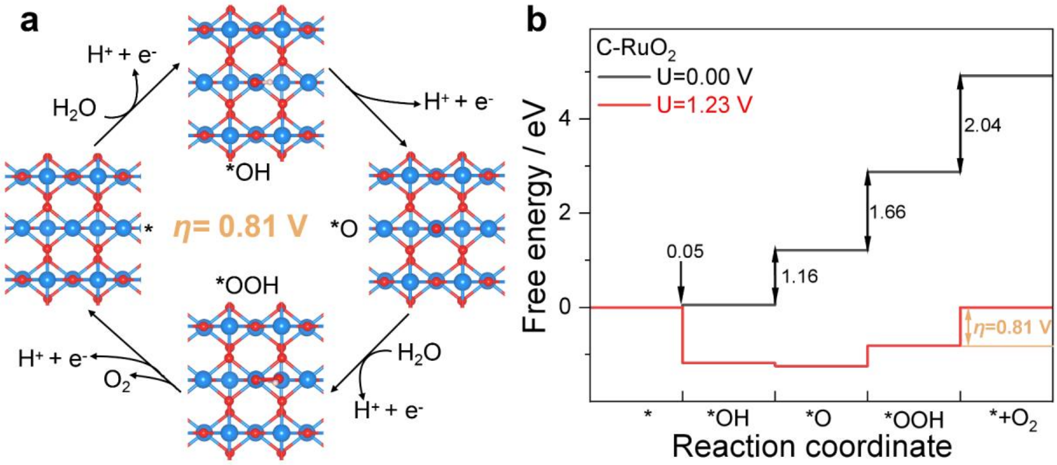

Supplementary Fig. 37 | Mechanism of OER steps and the free energy profile of OER over the C-RuO$_2$ catalyst. **a**, Schematic illustration of OER mechanism on the C-RuO$_2$ (blue, Ru; red, O; white, H). **b**, The reaction paths on C-RuO$_2$ catalyst with the set potential of 0 and 1.23 V. The η value indicates the rate of the catalyst determining the overpotential of the step.

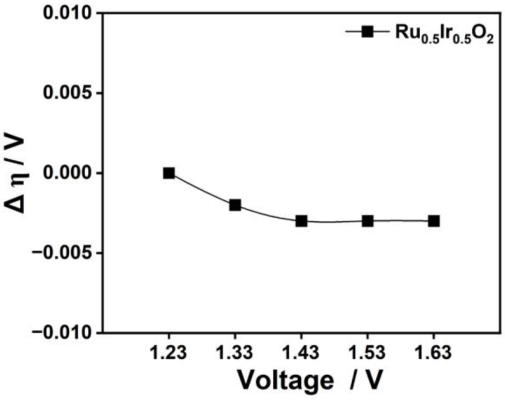

Supplementary Fig. 38 | The difference in OER overpotential (Δη, V) between jDFTx and VASPsol on $Ru_{0.5}Ir_{0.5}O_2$ under different applied voltage (V).

Supplementary Table 1 | The BET-based areas of $Ru_{0.5}Ir_{0.5}O_2$, C-IrO_2 and C-RuO_2 in this work.

**Catalysts**	**BET area / m^2 g^{-1}**
$Ru_{0.5}Ir_{0.5}O_2$	25.8
C-IrO_2	3.3
C-RuO_2	6.7

Supplementary Table 2 | ICP-AES analysis of $Ru_{0.5}Ir_{0.5}O_2$.

Catalyst	Ru content / wt %	Ir content / wt %	Ru content / at %	Ir content / at %
$Ru_{0.5}Ir_{0.5}O_2$	30.66	69.34	45.67	54.33
	32.58	67.42	48.32	51.68
	34.57	65.43	50.11	49.89

Note: The chemical composition ratio of $Ru_{0.5}Ir_{0.5}O_2$ is determined, and the optimal molar ratio of Ru is 0.5.

Supplementary Table 3 | XPS line shapes, binding energies and fitting parameters of Ru 3*p* and Ir 4*f* XPS spectra for Fig. 2b using CasaXPS software.

Catalysts	Peak	Position / eV	Area	FWHM / eV	Lineshape
$Ru_{0.5}Ir_{0.5}O_2$	Ru $3p_{3/2}$	461.6	53801.9	4.4	GL (30)
	Ru $3p_{1/2}$	483.6	25813.6	4.4	GL (30)
	Ir $4f_{7/2}$	62.2	15000.8	1.6	GL (30)
	Ir $4f_{5/2}$	65.2	12282.7	1.6	GL (30)
	Ir $4f_{7/2}$ (satellite 1)	63.6	5439.5	1.7	GL (30)
	Ir $4f_{5/2}$ (satellite 1)	66.6	4100.0	1.7	GL (30)
	Ir $4f_{7/2}$ (satellite 2)	67.5	6344.8	3.4	GL (30)
C-IrO_2	Ir $4f_{7/2}$	61.8	10762.0	1.26	GL (30)
	Ir $4f_{5/2}$	64.8	8554.1	1.26	GL (30)
	Ir $4f_{7/2}$ (satellite 1)	63.1	4775.2	1.6	GL (30)
	Ir $4f_{5/2}$ (satellite 1)	66.1	3523.8	1.6	GL (30)
	Ir $4f_{7/2}$ (satellite 2)	67.1	4457.2	3.2	GL (30)
C-RuO_2	Ru $3p_{3/2}$	462	14674.6	3.55	GL (30)
	Ru $3p_{1/2}$	484	7318.4	3.55	GL (30)

Note: FWHM, full width at half maximum.

Supplementary Table 4 | EXAFS fitting parameters at the Ru K-edge and Ir L_3-edge for various samples.

**Catalysts**	**Shell**	***CN***[a]	***R*(Å)**[b]	**σ^2(Å^2)**[c]	**ΔE_0(eV)**[d]	***R* factor**
			Ir L_3-edge (S_0^2=0.817)			
Ir foil	Ir-Ir	12*	2.71±0.01	0.0033±0.0001	8.2	0.0005
C-IrO_2	Ir-O	6.2±0.3	1.99±0.01	0.0027±0.0010	2.5	0.0068
	Ir-Ir	3.7±0.3	3.17±0.03	0.0052±0.0026	5.6	
	Ir-Ir	8.0±0.4	3.58±0.01			
$Ru_{0.5}Ir_{0.5}O_2$	Ir-O	5.9±0.3	2.01±0.01	0.0025±0.0002	1.3	0.0095
	Ir-Ru	1.7±0.4	3.14±0.01	0.0113±0.0016		
			Ru K-edge (S_0^2=0.85)			
Ru foil	Ru-Ru	12*	2.67±0.01	0.0043±0.0001	2.7	0.0057
C-RuO_2	Ru-O	6.0±0.2	1.96±0.01	0.0030±0.0005	-3.1	0.0081
	Ru-Ru	2.9±0.4	3.12±0.01	0.0030±0.0004		
	Ru-Ru	8.0±0.8	3.54±0.01	0.0030±0.0004		
$Ru_{0.5}Ir_{0.5}O_2$	Ru-O	3.0±0.7	2.07±0.01	0.0100±0.0021	10.4	0.0098
	Ru-Ir	1.5±0.2	2.83±0.02	0.0060±0.0029		

[a]*CN*, coordination number; [b]*R*, distance between absorber and backscatter atoms; [c]σ^2, Debye-Waller factor to account for both thermal and structural disorders; [d]ΔE_0, inner potential correction; *R* factor indicates the goodness of the fit. $S0^2$ was fixed to 0.817 and 0.805, according to the experimental EXAFS fit of Ir foil and Ru foil by fixing CN as the known crystallographic value. Fitting range: $3.0 \leq k$ (/Å) ≤ 17.9 and $1.0 \leq R$ (Å) ≤ 3.0 (Ir foil and Ru foil); $3.0 \leq k$ (/Å) ≤ 13.0 and $1.0 \leq R$ (Å) ≤ 4.0 (C-IrO_2); $2.0 \leq k$ (/Å) ≤ 16.9 and $1.0 \leq R$ (Å) ≤ 3.5 ($Ru_{0.5}Ir_{0.5}O_2$); $3.0 \leq k$ (/Å) ≤ 17.2 and $1.0 \leq R$ (Å) ≤ 4.0 (C-RuO_2); $2.0 \leq k$ (/Å) ≤ 12.0 and $1.0 \leq R$ (Å) ≤ 3.5 ($Ru_{0.5}Ir_{0.5}O_2$). A reasonable range of EXAFS fitting parameters: $0.700 < S_0^2 < 1.000$; $CN > 0$; $\sigma^2 > 0$ Å^2; $|\Delta E_0| < 10$ eV; *R* factor < 0.02.

Supplementary Table 5 | The ECSA of $Ru_{0.5}Ir_{0.5}O_2$, C-IrO_2 and C-RuO_2 in this work.

**Catalysts**	**ECSA / cm^2**
$Ru_{0.5}Ir_{0.5}O_2$	113.7
C-IrO_2	23.4
C-RuO_2	39.4

Supplementary Table 6 | Concentrations of Ru and Ir ions in electrolyte after the stability testing at 10 mA cm^{-2}.

Catalyst	Ru concentration / ppb			Ir concentration / ppb		
	**5 h**	**20 h**	**80 h**	**5 h**	**20 h**	**80 h**
**$Ru_{0.5}Ir_{0.5}O_2$**	0.3	5.1	22.7	0.09	1.6	7.1

Note: Chronopotentiometry measurement at 10 mA cm^{-2} with periodic sampling of the working electrolyte solution (5 mL of 20 mL) for the ICP-AES analysis and replacement of the aliquot with 5 mL of pure 0.5 M H_2SO_4 to keep the total electrolyte solution volume constant at 20 mL.

Supplementary Table 7 | The comparison of the OER performances (overpotential, TOF and mass activity) of $Ru_{0.5}Ir_{0.5}O_2$ and various reported catalysts.

**Electrocatalysts**	**Electrolyte**	**Overpotential[a] / mV**	**TOF / s^{-1}**	**Mass activity / A g_{Ru+Ir}^{-1}**	**Stability[b] / h**	**Reference**
**$Ru_{0.5}Ir_{0.5}O_2$**	**0.5 M H_2SO_4**	**151**	**6.84 @ 1.44 V vs. RHE**	**730.4 @ 1.44 V vs. RHE**	**618.3**	**This work**
C-IrO_2	0.5 M H_2SO_4	321	0.05 @ 1.44 V vs. RHE	6.6 @ 1.44 V vs. RHE	19.5	This work
C-RuO_2	0.5 M H_2SO_4	297	0.04 @ 1.44 V vs. RHE	5.6 @ 1.44 V vs. RHE	23.5	This work
$RuIrO_x$	0.5 M H_2SO_4	233	-	0.01 @ 1.35 V vs. RHE	24	*Nat. Commun.* **2019**, *10*, 4875
RuIr-NC	0.05 M H_2SO_4	165	-	796 @ 1.45 V vs. RHE	122	*Nat. Commun.* **2021**, *12*, 1145
Ru@IrO_x	0.05 M H_2SO_4	282	-	644.8 @ 1.56 V vs. RHE	24	*Chem* **2019**, *5*, 445-459
SrRuIr	0.5 M H_2SO_4	190	0.2 @ 1.53 V vs. RHE	654 @ 1.53 V vs. RHE	1500	*J. Am. Chem. Soc.* **2021**, *143*, 6482-6490
Co-RuIr	0.1 M $HClO_4$	235	-	-	25	*Adv. Mater.* **2019**, *31,* 1900510
RuIrTe NTs	0.5 M H_2SO_4	205	-	-	24	*J. Mater. Chem. A,* **2022**, *10,* 2021-2026
Ru_1-Pt_3Cu	0.1 M $HClO_4$	220	-	779 @ 1.51 V vs. RHE	28	*Nat. Catal.* **2019**, *2*, 304-313
12Ru/MnO_2	0.1 M $HClO_4$	161	0.33 @ 1.395V vs. RHE	1264 @ 1.395V vs. RHE	200	*Nat. Catal.* **2021**, *4*, 1012-1023
Ni-RuO_2	0.1 M $HClO_4$	214	-	-	20	*Nat. Mater.* **2022**, *22*, 100-108
$W_{0.2}Er_{0.1}Ru_{0.7}O_{2-\delta}$	0.5 M H_2SO_4	168	-	1518.6 @ 1.505 V vs. RHE	500	*Nat. Commun.* **2020**, *11*, 5368
a-$RuTe_2$ PNRs	0.5 M H_2SO_4	245	-	-	24	*Nat. Commun.* **2019**, *10*, 5692

Ru-N-C	0.5 M H_2SO_4	267	0.93 @ 1.497V vs. RHE	3571@ 1.497V vs. RHE	30	*Nat. Commun.* **2019**, *10*, 4849
$CaCu_3Ru_4O_{12}$	0.5 M H_2SO_4	171	-	-	24	*Nat. Commun.* **2019**, *10*, 3809
$Cr_{0.6}Ru_{0.4}O_2$	0.5 M H_2SO_4	178	0.15 @ 1.49V vs. RHE	229 @ 1.5 V vs. RHE	10	*Nat. Commun.* **2019**, *10*, 162
$Sr_{0.90}Na_{0.10}RuO_3$	0.1 M $HClO_4$	160	-	0.70 @ 1.35 V vs. RHE	-	*Nat. Commun.* **2019**, *10*, 2041
Cu-doped RuO_2	0.5 M H_2SO_4	188	0.0528 @ 1.48 V vs. RHE	-	8	*Adv. Mater.* **2018**, *30*, 1801351
RuO_2 NSs	0.5 M H_2SO_4	199	-	520 @ 1.46 V vs. RHE	6.3	*Energy Environ. Sci.* **2020**, *13*, 5143-5151
RuO_2 nanosheet	0.1 M $HClO_4$	255	-	10 @ 1.455 V vs. RHE	5.8	*Adv. Energy Mater.* **2019**, *9*, 1803795
Mn-RuO_2	0.5 M H_2SO_4	158	0.391 @ 1.48 V vs. RHE	596.38 @ 1.5 V vs. RHE	10	*ACS Catal.* **2020**, *10*, 1152-1160
$Co_{0.11}Ru_{0.89}O_{2-\delta}$	0.5 M H_2SO_4	169	-	-	50	*iScience* **2020**, *23*, 100756
Ru-RuO_2/CNT	0.5 M H_2SO_4	180	0.082 @ 1.63 V vs. RHE	-	30	*Nano Energy* **2019**, *61*, 576-583
$IrO_x/SrIrO_3$	0.5 M H_2SO_4	270-290	-	-	30	*Science* **2016**, *353*, 1011-1014
GB-$Ta_{0.1}Tm_{0.1}Ir_{0.8}O_{2-\delta}$	0.5 M H_2SO_4	198	2.54@ 1.868 V vs. RHE	3126@ 1.496 V vs. RHE	500	*Nat. Nanotechnol.***2021**,16, 1371-1377
6H-$SrIrO_3$	0.5 M H_2SO_4	248	-	75 @ 1.525 V vs. RHE	30	*Nat. Commun.* **2018**, *9*, 5236
Amorphous Ir nanosheets	0.1 M $HClO_4$	255	0.16 @ 1.53 V vs. RHE	221.8 @ 1.53 V vs. RHE	8	*Nat. Commun.* **2019**, *10*, 4855
1T-IrO_2	0.1 M $HClO_4$	197	4.2 @ 1.5 V vs. RHE	296.8 @ 1.5 V vs. RHE	40	*Nat. Commun.* **2021**, *12*, 6007
3R-IrO_2	0.1 M $HClO_4$	188	5.7 @ 1.5 V vs. RHE	691 @ 1.5 V vs. RHE	511	*Joule* **2021**, *5*, 1-14

Amorphous Li-IrO_x	0.5 M H_2SO_4	270	0.3 @ 1.53 V vs. RHE	5 @ 1.35 V vs. RHE	10	*J. Am. Chem. Soc.* **2019**, *141*, 3014-3023
IrO_2/GCN	0.5 M H_2SO_4	276	0.17 @ 1.6 V vs. RHE	1.3 @ 1.35 V vs. RHE	4	*Angew. Chem. Int. Ed.* **2019**, *131*, 12670-12674

a: The overpotentials required to achieve a current density of 10 mA cm^{-2};

b: Chronopotentiometric stability test at the current density of 10 mA cm^{-2} at room temperature.

Supplementary Table 8 | The Bader charges of $Ru_{0.5}Ir_{0.5}O_2$, C-IrO_2 and C-RuO_2 derived from the DFT calculations.

Catalysts	Element	Formal electron loss
$Ru_{0.5}Ir_{0.5}O_2$	Ru	1.47
	Ir	1.53
C-IrO_2	Ru	-
	Ir	1.52
C-RuO_2	Ru	1.52
	Ir	-

Supplementary Table 9 | The Ru-O and Ir-O bond length (Å) from DFT calculations for various catalysts.

Catalysts	Bond	Bond Length / Å	Bond Length / Å	Bond Length / Å	Bond Length / Å	Bond Length / Å	Bond Length / Å
C-IrO_2	Ir-O	1.965	1.965	1.958	1.958	1.958	1.958
$Ru_{0.5}Ir_{0.5}O_2$	Ir-O	2.008	1.995	1.984	2.035	2.006	2.030
	Ru-O	2.019	2.010	2.022	1.970	2.020	2.004
C-RuO_2	Ru-O	2.012	2.012	1.926	1.926	1.926	1.926

Supplementary Table 10 | The comparison of the OER performances (overpotential and estimated overpotential) of $Ru_{0.5}Ir_{0.5}O_2$ and various reported catalysts.

Electrocatalysts	Electrolyte	Overpotential[a] / mV	Estimated Overpotential / mV	Reference
**$Ru_{0.5}Ir_{0.5}O_2$**	**0.5 M H_2SO_4**	**151**	**360**	**This work**
Re-RuO_2	0.1 M $HClO_4$	190	790	*Nat. Commun.* **2023**, *14*, 354
$Li_{0.52}RuO_2$	0.5 M H_2SO_4	156	510	*Nat. Commun.* **2022**, *13*, 3784
$RuIrO_x$	0.5 M H_2SO_4	233	400	*Nat. Commun.* **2019**, *10*, 4875
$CaCu_3Ru_4O_{12}$	0.5 M H_2SO_4	171	660	*Nat. Commun.* **2019**, *10*, 3809
SrRuIr	0.5 M H_2SO_4	190	370	*J. Am. Chem. Soc.* **2021**, *143*, 6482-6490
$Ru_1Ir_1O_x$	0.5 M H_2SO_4	204	650	*Adv. Energy Mater.* **2021**, *11*, 2102883
3R-IrO_2	0.1 M $HClO_4$	188	550	*Joule* **2021**, *5*, 1-14
Ru_1-Pt_3Cu	0.1 M $HClO_4$	220	420	*Nat. Catal.* **2019**, *2*, 304-313
$W_{0.2}Er_{0.1}Ru_{0.7}O_{2-\delta}$	0.5 M H_2SO_4	168	530	*Nat. Commun.* **2020**, *11*, 5368
Ru-N-C	0.5 M H_2SO_4	267	590	*Nat. Commun.* **2019**, *10*, 4849
Cu-doped RuO_2	0.5 M H_2SO_4	188	660	*Adv. Mater.* **2018**, *30*, 1801351
RuO_2 NSs	0.5 M H_2SO_4	199	450	*Energy Environ. Sci.* **2020**, *13*, 5143-5151
RuO_2 nanosheet	0.1 M $HClO_4$	255	430	*Adv. Energy Mater.* **2019**, *9*, 1803795
Mn-RuO_2	0.5 M H_2SO_4	158	1480	*ACS Catal.* **2020**, *10*, 1152-1160

a: The overpotentials required to achieve a current density of 10 mA cm^{-2}.

Supplementary Table 11 | The comparison of the OER performances (overpotential and estimated overpotential) of C-RuO_2, C-IrO_2 and various reported rutile IrO_2 and RuO_2 catalysts.

Electrocatalysts	Electrolyte	Overpotential[a] / mV	Estimated Overpotential / mV	Reference
**C-RuO_2**	**0.5 M H_2SO_4**	**297**	**810**	**This work**
RuO_2	0.1 M $HClO_4$	258	890	*Nat. Commun.* **2023**, *14*, 354
RuO_2	0.5 M H_2SO_4	320	770	*Nat. Commun.* **2022**, *13*, 3784
RuO_2	0.5 M H_2SO_4	285	560	*J. Am. Chem. Soc.* **2021**, *143*, 6482-6490
RuO_x	0.5 M H_2SO_4	281	840	*Adv.Energy Mater.***2021***, 11*, 2102883
RuO_2	0.5 M H_2SO_4	316	850	*Nat. Commun.* **2019**, *10*, 3809
**C-IrO_2**	**0.5 M H_2SO_4**	**321**	**1000**	**This work**
IrO_x	0.5 M H_2SO_4	334	1020	*Adv.Energy Mater.* **2021***, 11*, 2102883
IrO_2	0.1 M $HClO_4$	406	680	*Joule* **2021**, 5, 1-14
IrO_2	0.5 M H_2SO_4	340	610	*J. Am. Chem. Soc.* **2021**, *143*, 6482-6490
IrO_2	0.5 M H_2SO_4	297	590	*Nat. Commun.* **2021**, *12*, 6007
IrO_2	0.5M H_2SO_4	397	580	*Adv. Sci.* **2022**, *9*, 2104636

[a] The overpotentials required to achieve a current density of 10 mA cm^{-2}.

Supplementary References

1 Mathew, K. *et al.* Implicit self-consistent electrolyte model in plane-wave density-functional theory. *J. Chem. Phys.* 151, 234101 (2019).

2 Mathew, K. *et al.* Implicit solvation model for density-functional study of nanocrystal surfaces and reaction pathways. *J. Chem. Phys.* 140, 084106 (2014).

3 Sundararaman, R. *et al.* JDFTx: Software for joint density-functional theory. *SoftwareX* 6, 278-284 (2017).

4 Perdew, J. P., Burke, K. & Ernzerhof, M. Generalized gradient approximation made simple. *Phys. Rev. Lett.* 77, 3865-3868 (1996).

5 Sundararaman, R. & Goddard, W. A., III. The charge-asymmetric nonlocally determined local-electric (CANDLE) solvation model. *J. Chem. Phys.* 142, 064107 (2015).
